# New Horizons in Near-Zero Refractive Index Photonics and Hyperbolic Metamaterials

**DOI:** 10.1021/acsphotonics.3c00747

**Published:** 2023-10-23

**Authors:** Michaël Lobet, Nathaniel Kinsey, Iñigo Liberal, Humeyra Caglayan, Paloma A. Huidobro, Emanuele Galiffi, Jorge Ricardo Mejía-Salazar, Giovanna Palermo, Zubin Jacob, Nicolò Maccaferri

**Affiliations:** † Department of Physics and Namur Institute of Structured Materials, University of Namur, Rue de Bruxelles 61, 5000 Namur, Belgium; ‡ John A. Paulson School of Engineering and Applied Sciences, Harvard University, 9 Oxford Street, Cambridge, Massachusetts 02138, United States; § Department of Electrical and Computer Engineering, Virginia Commonwealth University, Richmond, Virginia 23284, United States; ∥ Department of Electrical, Electronic and Communications Engineering, Institute of Smart Cities (ISC), Public University of Navarre (UPNA), Pamplona 31006, Spain; ⊥ Faculty of Engineering and Natural Science, Photonics, Tampere University, 33720 Tampere, Finland; # Departamento de Física Téorica de la Materia Condensada and Condensed Matter Physics Center (IFIMAC), Universidad Autónoma de Madrid, E-28049 Madrid, Spain; ○ Instituto de Telecomunicações, Instituto Superior Técnico-University of Lisbon, Avenida Rovisco Pais 1, Lisboa, 1049-001, Portugal; □ Photonics Initiative, Advanced Science Research Center, City University of New York, New York, New York 10027, United States; △ National Institute of Telecommunications (Inatel), Santa Rita do Sapucaí, 37540-000 MG, Brazil; ▽ Department of Physics, NLHT Lab, University of Calabria, 87036 Rende, Italy; ● CNR NANOTEC-Institute of Nanotechnology, Rende (CS), 87036 Rende, Italy; ■ Elmore Family School of Electrical and Computer Engineering, Purdue University, West Lafayette, Indiana 47907, United States; ▲ Birck Nanotechnology Center, Purdue University, West Lafayette, Indiana 47907, United States; ▼ Department of Physics, Umeå University, Linnaeus väg 24, 90187 Umeå, Sweden; ⬡ Department of Physics and Materials Science, University of Luxembourg, 162a avenue de la Faïencerie, L-1511 Luxembourg, Luxembourg

**Keywords:** near-zero refractive index photonics, hyperbolic metamaterials, nonlinear optics, sensing, time-varying photonics, thermal emission engineering

## Abstract

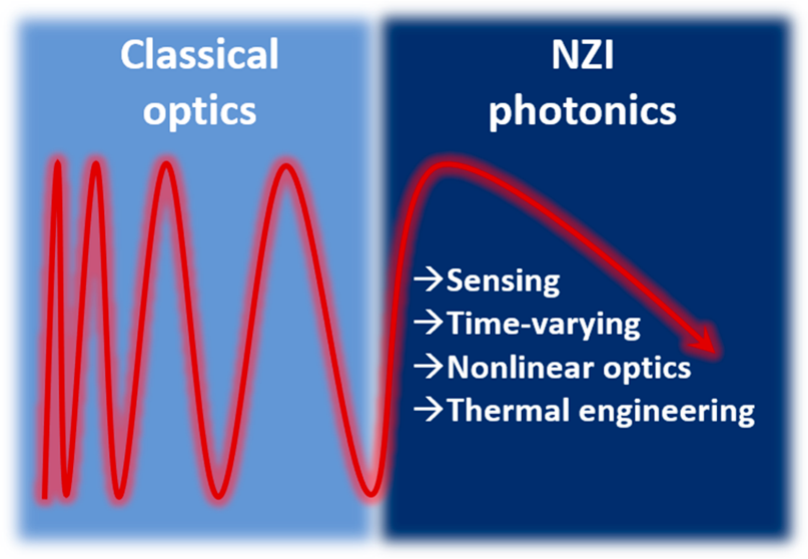

The engineering of the spatial and temporal properties of both the electric permittivity and the refractive index of materials is at the core of photonics. When vanishing to zero, those two variables provide efficient knobs to control light–matter interactions. This Perspective aims at providing an overview of the state of the art and the challenges in emerging research areas where the use of near-zero refractive index and hyperbolic metamaterials is pivotal, in particular, light and thermal emission, nonlinear optics, sensing applications, and time-varying photonics.

## Introduction

Generating, manipulating, and detecting light are essential actions in photonics that implicitly require interaction with materials. Tracing back to Maxwell’s equations, one can identify two physical quantities that are responsible for the interaction of electromagnetic waves with matter: the relative electric permittivity ε_r_ acting on the electric properties of matter, and its magnetic counterpart, the relative magnetic permeability μ_r_. Both quantities together give the material refractive index . Considering the wave-like nature of light picture, only a few variables are available in the photonics’ toolbox. One can either act on the refractive index contrast between materials, as a direct consequence of boundary conditions, or on the time/frequency dispersion of the refractive index. Therefore, over the past years, massive advances in the engineering of ε(*r⃗,t*), μ(*r⃗,t*) and *n*(*r⃗,t*) have been reported in photonics.^1−4^ From periodic spatial modulation of the index using photonic crystals^3,5,6^ and the simultaneous use of positive and negative permittivity in plasmonics,^2^ to the nanoscale engineering of the effective index that enabled negative values to be reached,^7^ control over constituent materials has unlocked new regimes of light–matter interactions. Here, we focus on near-zero refractive index (NZI) photonics^8−10^ and hyperbolic metamaterials (HMM).^11−17^ The current evolution, as well as new frontiers and future directions and challenges of these two correlated topics, are at the core of this Perspective.

**Figure 1:**
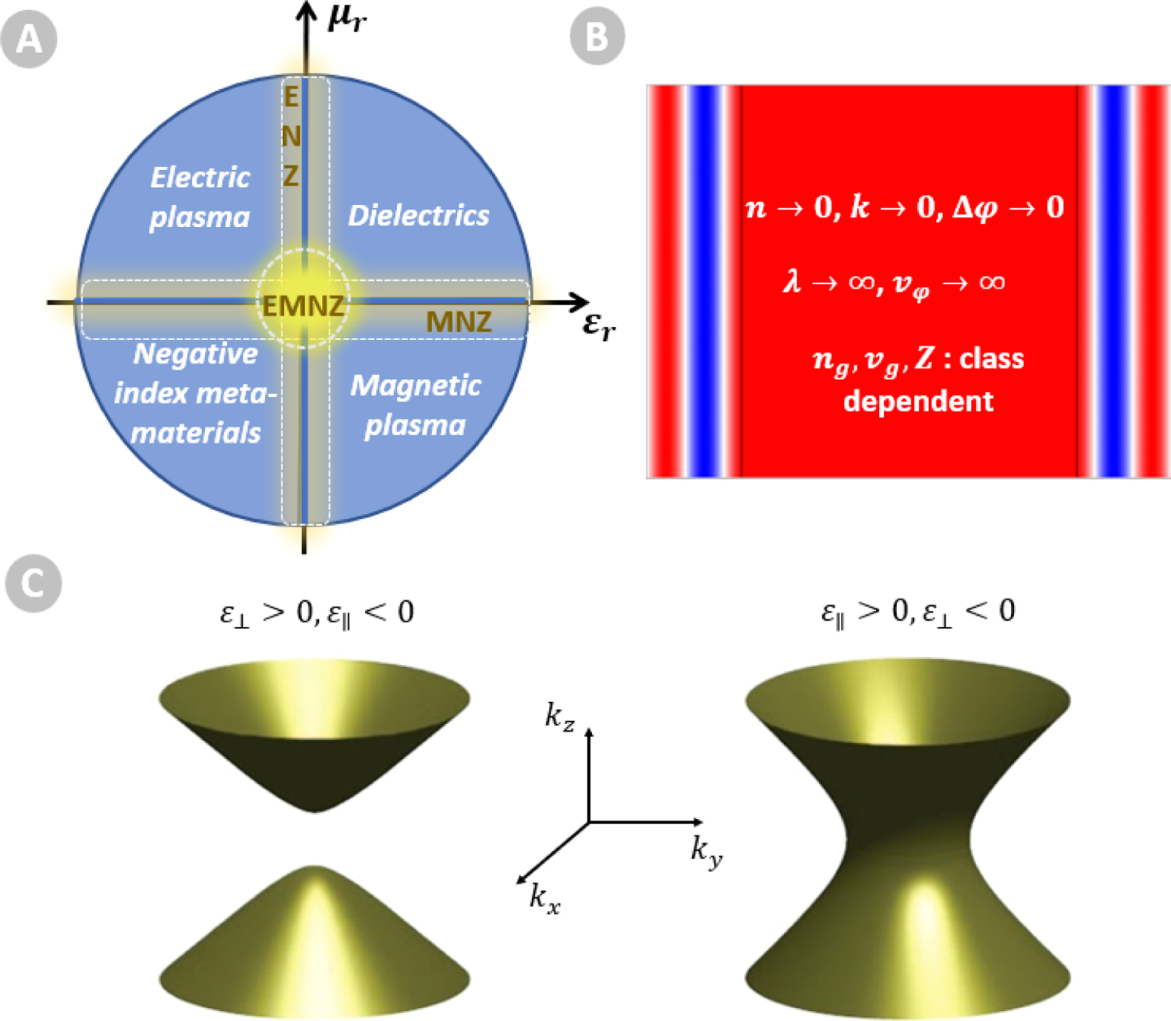
(a) Classification of photonic materials according to their relative electric permittivity ε_r_ and relative magnetic permeability μ_r_, exhibiting three NZI classes: ENZ class, MNZ class, and EMNZ class, inspired by refs (24 and 25). (b) Uniform phase distribution and electrodynamical quantities reaching extremes values in NZI media. (c) Isofrequency surfaces in HMMs. Reproduced with permission from ref (11). Copyright 2013 Nature.

While a new range of fabrication techniques has enabled the generation of a negative index, this is in principle possible only over a restricted set of frequencies. As a result, the index undergoes transitions between being positive and negative, opening frequency windows where the index is “near-zero”. As suggested by the provided definition of the refractive index in terms of its electric and magnetic constituent, the frequency range where the index has a near-zero response can be retrieved in three different ways (Figure 1a). The refractive index can reach zero by a vanishing electric permittivity, creating the epsilon-near-zero class (ENZ, ε → 0); by a vanishing magnetic permeability, inducing the mu-near-zero class (MNZ, μ → 0), or finally, by simultaneously vanishing permittivity and permeability, the epsilon-and-mu-near-zero class (EMNZ, ε → 0 and μ → 0).^8−10^ These three classes share common properties due to the vanishing index of refraction (Figure 1b), and we can refer to these materials as near-zero-index (NZI) materials. On the one hand, a range of physical quantities tend to infinity, such as the effective wavelength λ inside a NZI medium, , λ_0_ being the vacuum wavelength, and the phase velocity , with *c* being the fundamental constant defined as the speed of light in vacuum.^18^ On the other hand, some other quantities tend to zero, such as the wavevector *k* or the phase difference Δφ inside the NZI material, leading to a uniform phase distribution. Nevertheless, not all electrodynamical quantities either tend to zero or infinity in a NZI medium. Some quantities depend on the NZI class, i.e., the way one engineers the near-zero index response. For example, the wave impedance , the group velocity *v*_g_, or the related group index *n*_g_ = *c*/*v*_g_ present drastically different values according to the NZI class and their specific geometrical implementation.^19,20^ The ability to push multiple key parameters to the aforementioned extremes through NZI engineering enabled novel optical phenomena such as perfect transmission through distorted waveguides,^19^ cloaking,^21,22^ and inhibited diffraction.^23^

When investigating the transition of the relative permittivity around NZI frequency points, a particularly interesting situation led to the definition of hyperbolic metamaterials, which can be explained as follows. As briefly mentioned above, plasmonics opened a whole branch of photonics. A surface plasmon polariton (SPP) corresponds to a light-driven collective oscillation of electrons localized at the interface between materials with a dielectric (ε > 0) and metallic (ε < 0) dispersion. If the interface is flat, as in a thin layer, propagating SPP can propagate along the interface. Alternatively, if the interface has a closed shape, such as in a nanoparticle or a nanowire, the coherent electronic vibration is localized, and the excitation is referred to as a localized surface plasmon (LSPs). When multiple metal/dielectric interfaces supporting surface plasmons occur within subwavelength separation, the associated coupled electromagnetic field exhibits a collective response, which can be modeled by an effective medium approximation and the dispersion relation presents a unique anisotropic dispersion. More precisely, an effective permittivity tensor ε̂ can be derived such as
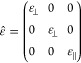
with ε_⊥_ (ε_∥_) the perpendicular (parallel) component with respect to the anisotropy axis, satisfying ε_⊥_ε_∥_ < 0. Consequently, their isofrequency surface presents a hyperbolic shape (Figure 1c).

Those materials, once predominantly engineered artificially, are referred to as hyperbolic metamaterials.^11,13,14,17,16^ However, they may occur naturally, too.^26−33^ It should be noted that one can engineer the permeability tensor μ̂ in a similar fashion, but this topic will not be covered in the present Perspective, which is structured as follows. We first highlight the impact NZI and HMMs photonics have recently had and are currently having on light and thermal emission. We then move to analyze NZI materials for nonlinear optics and all-optical switching, as well as sensing and magneto-optical applications. We conclude by focusing on the emerging NZI-based time-varying photonics. Overall, our aim is to provide a broad insight into the capabilities and challenges of using these engineered materials to manipulate light–matter interactions in both the frequency and time domain.

## Engineering of Light and Thermal Emission in NZI Media

### Quantum Radiative Transitions

**Figure 2:**
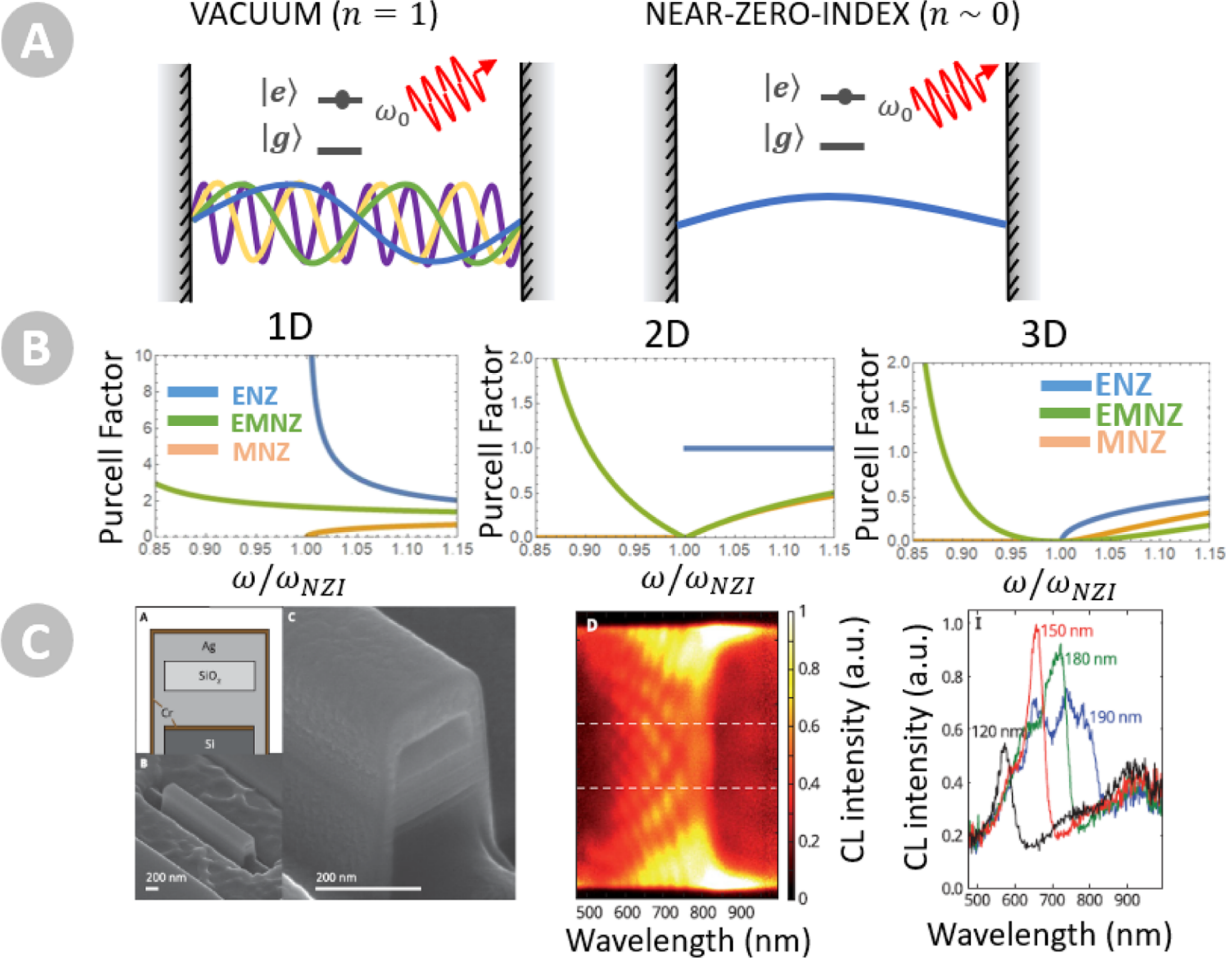
(a) Schematic depiction of a two-level system {|*e*⟩,|*g*⟩} with transition frequency ω_0_ coupled to a continuum of photonic modes in a virtual cavity model both in (left) vacuum and in (right) a near-zero-index (NZI) medium that suppresses the spatial density of modes. (b) Purcell factor, PF = Γ_s_/Γ_0_, in one-dimensional (1D, left), two-dimensional (2D, center), and three-dimensional (3D, right) systems, mimicking NZI media with ENZ, MNZ, and EMNZ material properties. ω_NZI_ refers to the near-zero refractive index frequency crossing. Reproduced with permission from ref (20). Copyright 2020 ACS. (c) (Left) SEM image of a rectangular metallic waveguide effectively implementing a 1D ENZ medium at optical frequencies. (Center) Cathodoluminiscence (CL) intensity as a function of wavelength and emission point demonstrating position-independent properties at the effective ENZ wavelength. (Right) CL intensity for different waveguide widths confirming the emission enhancement at the ENZ wavelength. Reproduced with permission from ref (36). Copyright 2013 APS.

NZI media have a profound and nontrivial impact on quantum radiative transitions, e.g., spontaneous emission, stimulated emission, and absorption. Intuitively, one can link the rate of a radiative process with the local density of optical states (LDOS). Then, since a NZI condition implies a depletion of the space of the optical modes (Figure 2a), one would be tempted to conclude that NZI media inhibits all radiative transitions, like in the band gap in a photonic crystal. However, this intuitive picture can be misleading. Because the coupling strength also scales with the refractive index, it turns out that a variety of nontrivial radiative phenomena can be observed in the zero-index limit, both as a function of the class of NZI media (ENZ, MNZ, EMNZ) and its effective dimensionality *D* (3D, 2D, 1D). Specifically, the spontaneous emission decay rate Γ_s_, normalized to its free-space counterpart Γ_0_, scales as follows^20^
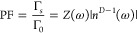


This equation must be evaluated when the transition frequency of the emitter ω lies in a propagating regime, where both the medium impedance *Z*(ω) and the refractive index *n*(ω) are real. It illustrates also how a variety of effects can be observed as the refractive index approaches zero (Figure 2b). For example, in three-dimensional media (*D* = 3), the decay rate vanishes independently of the class of NZI media, following the intuition that in NZI media the space of optical modes is depleted. However, a finite decay rate is obtained for 2D ENZ media and 1D EMNZ media, and the decay rate diverges in 1D ENZ media. The equation above assumes that the emitters are directly coupled to NZI modes, which is an accurate assumption only for some configurations. Nonetheless, when an emitter is immersed in a continuous medium, one should be careful on accounting for the coupling to the environment, e.g., with the inclusion of local cavity models. The complex interaction of the quantum emitter with surrounding boundaries can lead to further inhibition^34^ or enhancement^35^ effects. Therefore, very rich emission phenomena arise in NZI media as a function of the class of NZI medium, dimensionality, and how the emitter is coupled to the environment. At the same time, experimental studies of these effects are still rising.

1D ENZ media have been experimentally demonstrated at optical frequencies by using metallic rectangular metallic waveguides.^37,36^ These experiments have also confirmed both photoluminescence^37^ and cathodoluminescence^36^ enhancements, exemplifying how 1D ENZ media enhances radiating transitions, even in a photonic environment depleted of optical modes. Interestingly, the experiment in ref (36) also demonstrated position-independent emission, confirming how the enlargement of the wavelength can reduce the accuracy requirements in positioning quantum emitters (Figure 2c).

Engineering spontaneous emission also opens new opportunities for lasing. A photonic crystal laser with parameters compatible with 2D EMNZ media presents a Dirac cone at the Γ point of the Brillouin zone.^38^ Their laser is single-mode and remains so as the size of the cavity increases while usually many-order modes appear with increasing size. They suggest that the scale-invariant property of the cavity is related to the uniform phase property of the NZI environment. The impact of the NZI environment on light emission is thus an interesting direction for the coming future, especially for designing low-threshold lasers^39^ or superradiant lasers.^40^

### Applications in Quantum Technology

Describing spontaneous emission through a decay rate intrinsically assumes operating in the weak coupling regime and/or under the Markovian approximation.^4^ In the weak regime, the emission dynamics follow a simple exponential decay, which can be described by a single parameter, the decay rate or lifetime. However, as NZI frequency points typically take place at the edge of a band gap (or when a band gap is closed), a wider collection of decay effects can be observed in the nonperturbative regime.^41^ In this regime, the decay dynamics can be arbitrarily complex, giving access to a wider range of physical phenomena such as the saturation of the decay rate at a band-edge, the excitation of long-lived bound states, and fractional decay dynamics via the contribution of branch-cut singularities.^41^ The importance of these effects, and the interference between them, can be tuned by the design of the shape and size of NZI nanostructures. Interestingly, the possibility of accessing different classes of decay and interaction channels is a convenient tool for quantum simulation, where different physical systems can be implemented and tuned as a function of the dominant nonperturbative decay mechanism.^42^

Beyond modifying the individual decay properties of a single emitter, the enlargement of the wavelength in NZI media can trigger collective effects in ensembles of quantum emitters. Thus, NZI media act as optical reservoirs for quantum emitters, which could increase the interaction between optical fields and quantum systems and exhibit enhanced energy transfer and efficient inter-emitter interactions. Several numerical studies have highlighted that NZI media can facilitate the observation of collective effects, such as superradiance,^43,44^ and provide new strategies for entanglement generation.^45−49^

Moreover, the concept of entanglement, or nonseparability, between qubits is important in various quantum processes such as quantum cryptography and teleportation. While entanglement has traditionally been observed in systems of atoms and ions, it is becoming increasingly accessible in other areas of quantum physics. Specifically, short-distance entanglement has been observed in quantum dots, nanotubes, and molecules, but long-range, i.e., for distances longer compared to the wavelength of light,^50,51^ qubit–qubit interactions are necessary for long-distance information transfer. In this context, NZI waveguides might represent a game changer due to their aforementioned peculiar properties. As examples, numerical studies^45−49^ showed that ENZ media outperform the subwavelength distance limitations of qubits cooperative emission in a homogeneous medium. These studies adopted ENZ waveguides into quantum systems, which can be relevant in generating distinctive optical sources, robust entangled states, and other innovative optical applications in different fields of study. It is worth mentioning here that typically electron–phonon, ohmic, and inherent losses of the excited ENZ mode, as well as propagation losses, contribute to the transient nature of qubits entanglement mediated by an ENZ medium. Also, the qubit–qubit dissipative coupling induces modified collective decay rates, i.e., superradiant Γ + Γ _12_ and subradiant states Γ – Γ_12_, which exhibit pure superradiant emission when the Γ = Γ_12_ condition is satisfied.^52^ Here, Γ is the decay rate of the individual emitters, while Γ_12_ is the modification of the decay rate due to coupling. In summary, the long-range quantum entanglement between a pair of qubits mediated by an ENZ waveguide persists over extended periods and long distances. Thus, it is possible to obtain a robust entanglement of qubits coupled to the ENZ waveguide channel.

Similar to spontaneous emission, NZI media affects other quantum radiative transitions and light–matter interactions. This is particularly exciting for quantum technologies, since achieving strong light–matter coupling in solid-state systems is required for the design of scalable quantum devices. Along this line it was recently found that dispersion engineering around the ENZ frequency strengthens magnon–photon coupling.^53,54^ Strong opto-magnonic coupling would allow for quantum state transfer in hybrid quantum systems. This is a recent and promising direction for NZI materials, and both fundamental and practical implementation advances will be needed to assess the technological potential of NZI media for opto-magnonics.

### Energy vs Momentum Considerations

Light–matter interactions are usually described through energetic considerations. However, as noted by Einstein in his seminal work,^55,56^ momentum deserves equal theoretical attention due to its conservation property. Examining light–matter interactions inside NZI materials from a momentum perspective,^57^ therefore, offers a different picture. Closely related to the Abraham–Minkowski debate,^58−60^ light momentum is nontrivial to define. On one hand, Barnett^61^ associated Minkowski’s momentum to the canonical momentum, which is closely correlated to the wave-like nature of light and to the phase refractive index.^62^ On the other hand, the Abraham momentum is connected to the kinetic momentum and a particle description of light, represented in equations by the group index. Due to the vanishing index of refraction, NZI induces a vanishing Minkowski momentum. Inhibition of fundamental radiative processes inside 3D NZI can be understood as the impossibility to exchange momentum inside such media.^20^ Similarly, diffraction by a slit, which can be seen as a momentum transfer in the direction orthogonal to light propagation is also inhibited.^23^ It would be an interesting perspective to generalize these momentum intuitions to other dimensionalities of NZI materials,^20^ especially in the case of the enhanced light–matter interactions in 1D ENZ, as described above. Moreover, as pointed out by Kinsey,^63^ the developed momentum framework could be applied to space–time nonlinear interactions presenting strong spatial and temporal changes. The intriguing regime of these nonlinear responses could benefit from momentum considerations.

### Thermal Emission in NZI and HMM Media

Thermal emission is another radiative process of fundamental relevance, which historically was the first to motivate a quantum theory of light. Moreover, thermal emission is also a key process in multiple technologies such as heat and energy management, sensing and communications. However, thermal emission is broadband, temporally incoherent, isotropic, and unpolarized, which makes it difficult to control and manipulate. Therefore, different nanophotonic technologies attempt to change these properties by using nanostructured gratings, resonators and/or complex metamaterials.^64−66^ Again, because the wavelength is effectively stretched in a NZI medium, it was theoretically demonstrated that the spatial coherence of thermal fields is intrinsically enhanced in NZI media.^67^ This interesting result poses a new perspective in engineering thermal emission, where one can enhance the spatial coherence of thermal fields, without the need to resorting to complex nanofabrication processes.^67^ In fact, the intrinsic enhancement of thermal emission in ENZ and epsilon-near-pole (ENP) substrates was highlighted by early works in the field of HMM.^68^ Hyperbolic media adds a layer of complexity around the ENZ frequency points, resulting in optical topological transitions, where thermal emission can be selectively enhanced or suppressed.^69^

Since the medium impedance is enlarged as the permittivity approaches zero, ENZ media naturally acts as high-impedance surface^70^ or artificial magnetic conductor.^71^ As the tangential electric fields double their strength near a high-impedance surface, ENZ substrates intrinsically enhance the interaction with ultrathin metallic films. Several prototypes of ultrathin metallic film thermal emitters have been demonstrated using this principle.^72,73^ Moreover, since extreme boundaries are an intrinsic property of NZI media, these emitters have the technological advantage of not requiring from complex nanofabrication processes, and presenting narrowband but spectrally stable emission lines.^72,73^

## Nonlinear Properties of NZI Media and Their Application to All-Optical Switching

Optical switching via nonlinear index modulation has long been a goal of the field, driven by the promise of all-optical devices that are exceptionally fast and operate in environments where electrical control may not be feasible. Through advancements in materials, applications such as saturable mirrors for passive mode-locking,^74−76^ laser protective eyewear,^77,78^ and bistable devices^79,80^ just to name a few, have been realized, alongside the continual quest to pursue all-optical logic devices.^81−83^ For these operations to perform well, devices must effectively modify reflection/transmission/absorption and demonstrate either a latching temporal response or an ultrafast (ideally THz) response, depending on the use case. Bearing in mind these considerations, we can turn our attention to the recent developments in ENZ materials and nonlinear optical interactions to consider the advantages and challenges of using ENZ in this context.

**Figure 3:**
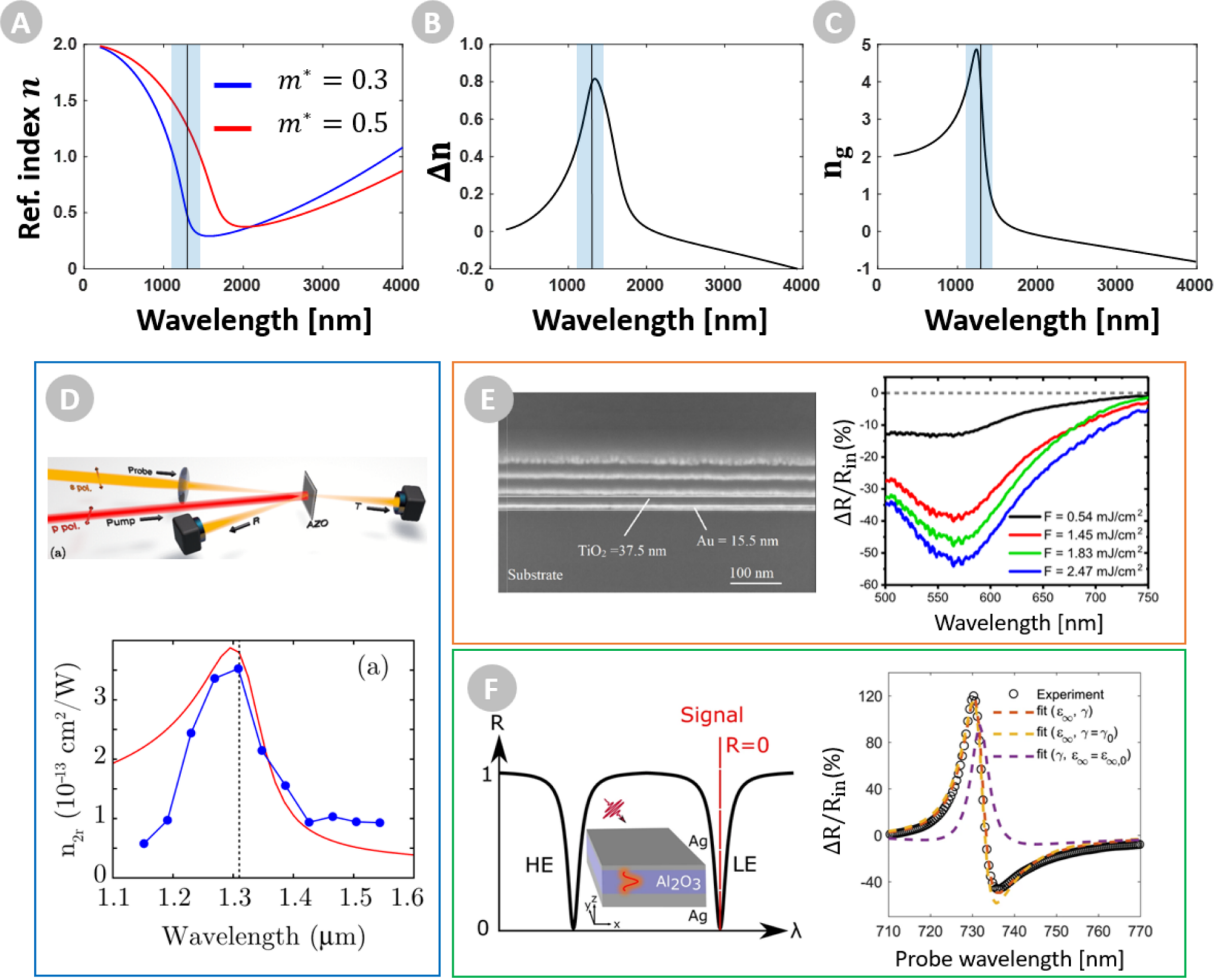
(a) Real part of the refractive index of a Drude-based material (blue) with ε_∞_ = 4, τ = 6 fs, *N* = 8 × 10^20^ cm^–3^, whose effective mass m* is modulated via intraband nonlinear processes, resulting in a shift of the index curve (red), giving rise to a (b) change in refractive index. (c) Group index of the unmodulated Drude-based film, as shown in (a). The ENZ region is shaded blue, with the crossover wavelength indicated as a vertical line. (d) Strong index tuning in Al:ZnO films with ENZ near 1300 nm. Reproduced with permission from ref (94). Copyright 2016 APS. (e) Strong modulation of transmission in effective ENZ materials with crossover at 509 nm. Reproduced with permission from ref (96). Copyright 2020 APS. (f) Modulation of cavity reflection for the guided plasmonic mode, with a mode index near zero. Reproduced with permission from ref (97). Copyright 2020 Nature.

For homogeneous materials, ENZ effects are generally achieved by introducing free carriers, for example, by degenerately doping a semiconductor (e.g., Al:ZnO, In:Sn_2_O_3_). In this case, the ENZ condition significantly modifies the dispersion of the material, facilitating strong changes in index even when far from a material resonance (Figure 3a,b) where there may otherwise be minimal dispersion. In this view, ENZ falls into the class of slow-light enhancement schemes for nonlinear optics^84−87^ (*n*_g_∼ 2–10 for popular ENZ oxides,^88^ see Figure 3c), where adding dispersion is used to generate increased light–matter interaction. The nonlinearity in ENZ arises from the modification of the index dispersion either through free-carrier generation (interband effect, blue-shift of index curve) and free-carrier redistribution (intraband effect, red-shift of index curve, see the following for more information).^89−92^ ENZ simultaneously improves the absorption of the excitation and provides a steep change in index at a given frequency, which has been shown to facilitate large index modulation on the scale of 0.1–1 with ∼1 ps relaxation times (Figure 3d–f).^93−95^

To place the performance of ENZ in context, we can compare the nonlinear coefficients to other materials. But before beginning to make this comparison, it is important to note that variations in the fundamental material and experimental conditions make absolute comparisons a great challenge. As a result, the following is intended to provide a general view on the order of magnitude of responses and trade-offs rather than the specific performance of any given material. Additionally, because nonlinearities in ENZ are non-instantaneous and involve real states (so-called “slow” processes), they should not be compared to instantaneous nonlinearities involving virtual states (so-called “fast” processes), as is common, as they are well-known to be much larger.^89,98^ A more appropriate comparison is to similar non-instantaneous process materials, such as semiconductors and metals. Finally, while it is common to quantify nonlinearities via χ^(3)^, *n*_2_, or α_2_, these terms imply properties such as linearity with respect to applied irradiance and an instantaneous response. Such properties are not valid assumptions for the “slow” nonlinearities in ENZ materials. Thus, we denote the quantities as χ^(3)^_eff_, *n*_2,eff_, or α_2,eff_, where subscript “eff” denotes an effective Kerr-like modulation to the optical properties, to highlight that these coefficients do not obey the same rules and depend greatly on properties such as pulse width, applied irradiance, angle of incidence, film thickness, etc.

Now, for ENZ oxides such as Al:ZnO, Ga:ZnO, and In:Sn_2_O_3_, *n*_2,eff_ = Δ*n*/*I* ∼ 0.1–5 × 10^–3^ cm^2^/GW (see Table 1) for 1100–1700 nm with relaxation on the order of ∼1 ps, depending on the wavelength(s) employed.^102,104^ This can be compared to free-carrier nonlinearities in the same spectral region for the GaAs platform where *n*_2,eff_ ∼ 0.1–0.3 × 10^–3^ cm^2^/GW (see Table 1) with response times of ∼1 ns (crystalline GaAs)^100^ that can be reduced to ∼1 ps for low-temperature grown GaAs.^105^ Thus, under optimal excitation conditions, nonlinearities in ENZ oxides provide up to an order of magnitude increase in the strength of the nonlinearity at normal incidence while improving upon the speed. For more information on nonlinear coefficients of various ENZ materials, see ref (106). It is important to note here that a comparison with virtual processes (for example, in semiconductors, off-resonance or dielectrics like SiO_2_) are not appropriate, as the mechanisms of the nonlinearity are different, and real effects are known to be much larger than their virtual counterparts.

**Table 1:** Epsilon-near-Zero *n*_2,eff_ Coefficients with Associated Experimental Parameters^a^

material	*n*_2,eff_ (cm^2^ GW^–1^)^b^^,^^c^	relaxation (ps)	excitation λ (nm)	probe λ (nm)	crossover λ (nm)	pulse width (fs)	technique
Si^99^	4.5 × 10^–5^	n/r	1540			130	Z-scan
GaAs^100^	3 × 10^–4^	n/r	1680			111	Z-scan
AZO^94^	3.5 × 10^–4^	∼0.8	785	1258	∼1300	100	R/T
ITO^101^	1.80 × 10^–3^	∼1	1100	1250	∼1200	150	B.D.
GZO^102^	5 × 10^–3^	∼1	1620	1700	1710	60	R/T
Au-TiO_2_^96^	1.2 × 10^–2^	∼8	470	610	605	120	R/T
Ant.-ITO^103^	–3.7	∼1	1240		1240	140	Z-scan

a Variations between AZO, GZO, and ITO are largely due to experimental parameter selection (e.g., pump/probe wavelengths) rather than differences in the underlying material.

b Note all the values are taken for near normal incidence beams.

c Note that nonlinear index coefficients are functions of the excitation-probe wavelengths, pulse width, sample thickness, irradiance, and angle of incidence. Care should be taken when attempting to use the values outside of the experimental conditions used.

While a useful gain, the introduction of ENZ to modify the dispersion of thin films, does not result in a radical performance jump when compared to existing platforms. Additionally, optical loss (due to free carriers) was introduced. As a result, ENZ devices suffer a limited size and must contend with thermal build-up/dissipation that must be addressed to realize a high-frequency operation.^107−110^

Although the fundamental gains in nonlinearity may not have been extreme, it is important to point out that the primary price paid is loss. In scenarios where devices are small, such loss may not be a large factor in performance (although thermal dissipation remains a concern). As a result, the use of the ENZ region to tailor the dispersion of a material is able to provide an order of magnitude increase in the nonlinearity over competing materials while maintaining a fast operation, a quite large bandwidth (∼400 nm) in the highly relevant telecommunications spectrum, and with readily available materials whose properties can be easily tuned during growth.^9^ Additionally, a key benefit of the ENZ oxides is their impressive damage threshold. Routinely, experiments utilize irradiance levels of 10 to 1000 GW/cm^2^ without permanent damage to the film.^91,94,95,106^ This allows ENZ to achieve large absolute changes in the refractive index (Δ*n* ∼ 0.1–1), despite only a marginally improved *n*_2,eff_ value and, consequently, the large absolute changes to reflection, transmission, and absorption at normal incidence that have been observed. With this view, the question becomes how can we push the strength of the base nonlinearity (*n*_2,eff_) further to mitigate the need for such high irradiance levels? While gains are predicted when shifting ENZ to the mid infrared using lower-bandgap materials with lower doping levels,^89,111^ the tried-and-true method of adding structure is one avenue to continue to engineer the dispersion and improve nonlinear interactions.^112−115^ This can be done by structuring the base material (such as forming nanoresonators, i.e., meta antennas), coupling the material with a structured layer (such as plasmonic antennas)^116−120^ or by mixing multiple materials to achieve an effective ENZ property.^96,121−123^ In general, these approaches allow additional freedom to control the dispersion of the device by introducing resonance(s), anisotropy, or both. Recent efforts include coupling to ENZ/Berreman/plasmonic modes within thin layer(s),^121,124−128^ incorporating resonant metallic nanoantennas on top of an ENZ layer,^103,129,130^ and utilizing layered metal-dielectric stacks to produce an effective ENZ condition.^96,131^ These approaches have been successful in reducing the irradiance required to achieve strong control over nonlinear interactions to ∼1–10 GW/cm^2^ (a 10–100x reduction), as well as transitioning ENZ into the visible region where natural ENZ materials, such as the doped oxides, are unable to reach. However, these gains are not free. From our view of dispersion engineering, the introduction of structure incurs an additional price of reduced bandwidth (10–100 nm), may also require specific excitation conditions (e.g., specific angles of incidence or wavelengths), can lengthen the relaxation time due to nonlinear processes in the added material (e.g., 5–10 ps recovery in metals^132^), and add overall complexity. In total, these undercut some of the key strengths of the ENZ condition, whose ultimate practicality depends on the constraints of a particular application.

In summary, the ENZ condition provides several unique benefits to the nonlinear space founded in the control over material dispersion and also brings baggage in the form of optical loss and only a moderate enhancement. As such, it is not a straightforward solution to the challenges facing nonlinear applications and must be employed appropriately. The primary question facing the community is whether the benefit of ENZ can overcome its limitations and impact an application of relevance. While recent efforts have suggested avenues in pulse characterization,^133^ frequency shifting,^88,134−136^ bistable devices,^137,138^ and THz generation,^139,140^ the work is ongoing. We see potential benefits in areas where control over high irradiances is needed or in scenarios where narrow operating bandwidths are utilized, as well as in the use of weakly resonant structures, such as plasmonic antennas, to provide a middle ground wherein the operational spectral bandwidth can remain reasonably broad (∼100 nm) while gaining additional improvement to the nonlinearity.

## HMM and ENZ for Sensing Applications

**Figure 4:**
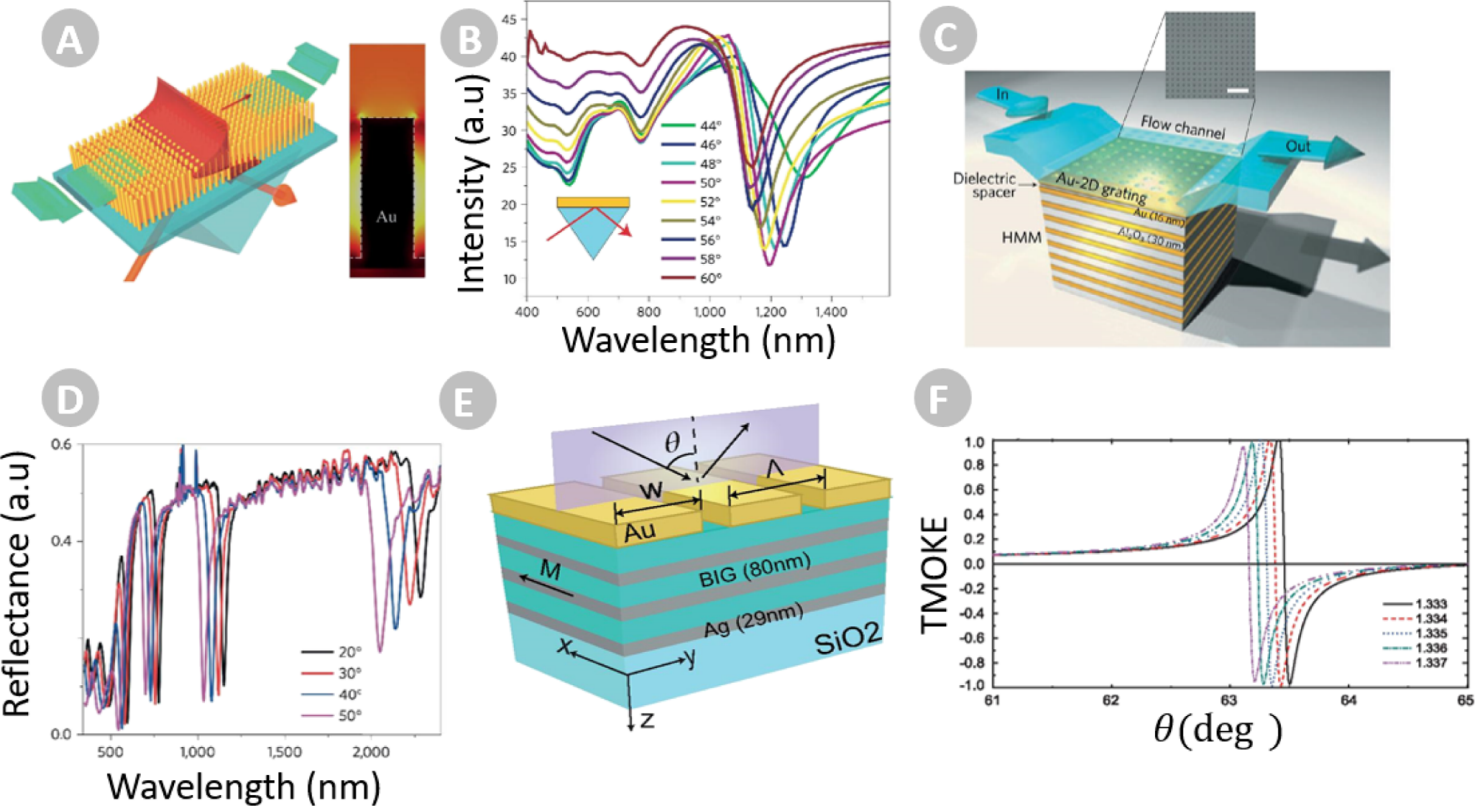
(a) Schematic of a conventional Kretschmann-like setup for plasmonic nanorod HMM biosensors and (b) their corresponding reflectance curves for different incident angles. Reproduced with permission from ref (144). Copyright 2009 Nature. The inset in (a) shows the electromagnetic field confinement in the volume of the nanorod array. Reproduced with permission from ref (145). Copyright 2022 OPG. (c) Illustration of a grating-coupler-based multilayer HMM biosensor with a fully integrated fluid flow channel. The inset shows a scanning electron microscopy image of the subwavelength gold diffraction grating on top of the HMM. (d) The reflectance spectra for the grating-coupler-HMM at different angles of incidence. Reproduced with permission from ref (146). Copyright 2016 Nature. The blue shift of resonance angles in (b) and (d) with increasing angle of incidence demonstrate that the VPP modes are guided modes. (e) Pictorial view of a MO-HMM comprising dielectric MO layers of bismuth–iron garnet (BIG) and Ag. (f) Fano-like TMOKE curves for the magnetoplasmonic structure in (e) when varying the superstrate refractive index from 1.333 to 1.337. Reproduced with permission from ref (149). Copyright 2022 ACS.

The unusual optical properties of HMM have proven to be useful for optical biosensors with unprecedented levels of sensitivity and resolution.^141−143^ Two prototypical HMM systems, comprising plasmonic nanorod arrays^144,145^ and plasmonic/dielectric multilayers,^146^ are illustrated in Figure 4a and c, respectively. These nanostructures support the so-called volume plasmon polariton (VPP) resonances, which are guided modes resulting from collective excitations of plasmonic resonances in the constituent multilayers^147,148^ or nanorods.^144,145^ In contrast to conventional surface plasmon polaritons (SPPs), VPPs have their associated electromagnetic fields largely concentrated in the volume of the metamaterial slab and decay exponentially in the superstrate region.^144,146,148^ The latter is demonstrated for the nanorod array in the inset of Figure 4a, where simulations of the near-field profile (under VPP resonance) around a single nanorod are shown. This unique feature has inspired two different mechanisms for biosensing applications. First, instead of using continuous flat films, the surfaces of the nanorods can be functionalized with bioreceptors to greatly increase the surface area in contact with the analyte region, producing sensitivity (*S* = Δλ/Δ*n*) values even higher than 40000 nm/RIU (refractive index unit).^144,145^

Nevertheless, the detection mechanism of plasmonic nanorod metamaterials requires the use of a Kretschmann-like setup, hindering miniaturization due to the need to use bulky prism couplers. Furthermore, plasmonic nanorod metamaterials exhibit a single and relatively broad VPP resonance in the infrared region, as can be observed from Figure 4b, which also limits the resolution levels. The second biosensing approach considers highly integrable grating-couplers for the excitation of VPPs in plasmonic/dielectric multilayer HMM.^146^Figure 4d shows that various VPP resonances, ranging from infrared to visible wavelengths, are allowed in the multilayer HMM. Some of these resonance dips are narrower than the ones for nanorod metamaterials, yielding higher values for the figure-of-merit  (where Δλ, Δ*n*, and Δω are the resonance shift, refractive index change, and full-width of the resonant dip at half-maximum), but with lower sensitivity (*S* < 30000 nm/RIU).^146^ A recent proposal combined the advantages of both HMM biosensor configurations into a single structure (by using nanocavities in a multilayer HMM,^150^ achieving detection limits down to the zeptomole range (i.e., a few tens of molecules).

Despite these breakthroughs, there are still challenges that need to be overcome. For example, the intrinsic ohmic losses of metallic inclusions induce wide resonance curves with large overlaps, which limits resolution when working with ultralow molecular weight analytes. In addition, biodetection is limited to achiral analytes, making it necessary to use fluorescence-enhanced biosensing techniques for the detection of chiral biomolecules.^150^ Attempts to surpass these drawbacks include HMMs interfaced with chiral metasurfaces,^151^ new concepts for manufacturing hyperbolic,^116,152,153^ and ENZ metamaterials,^154^ as well as the fabrication of magneto-optical (MO) and/or magnetically-active HMMs.^118,155−159^ In MO-HMMs one can take advantage of the transverse MO Kerr effect (TMOKE), with sharp Fano-like curves, to enhance the resolution levels of HMM-based biosensors,^149^ following a similar approach previously introduced using magnetic nanostructures.^160−166^ To illustrate the last mechanism, we consider the grating coupled MO-HMM in Figure 4e, composed by alternating layers of dielectric MO material (BIG in this case) and Ag. Instead of using the reflectance curves (as in conventional non-MO HMM), we may use the TMOKE (as seen from Figure 4f) to reach FOM values as high as 840. In comparison to conventional HMM, achieving FOM up to 590, the use of MO-HMM enables a way to obtain highly enhanced resolution for biosensing applications. Furthermore, computer-aided optimization of the sensor design can be performed with artificial intelligence algorithms, which may not only improve resolution, but also the sensitivity of MO-HMM nanostructures.^167^

## ENZ Media for Time-Varying Photonics

The possibility of temporally modulating the optical properties of matter via ultrafast optical pumping is establishing a new paradigm for enhanced wave control.^168^ While static nanophotonic platforms obey energy conservation and reciprocity, time-modulated systems can overcome these bounds, enabling new functionalities such as nonreciprocity,^169−174^ frequency generation^175^ and translation,^176,177^ time-diffraction,^178^ the engineering of photonic gauge fields,^179^ and synthetic frequency dimensions,^180^ as well as photonic Floquet matter,^181,182^ among others. While the field has witnessed dramatic progress at low frequencies, leading to, e.g., the first observation of photonic time-reflection^183^ and temporal coherent wave control,^184^ the prospect of unlocking this new wave-control paradigm at near-visible frequencies represents a unique opportunity to broaden and deepen the impact horizon amidst the current rise of photonic technology.^185^

**Figure 5:**
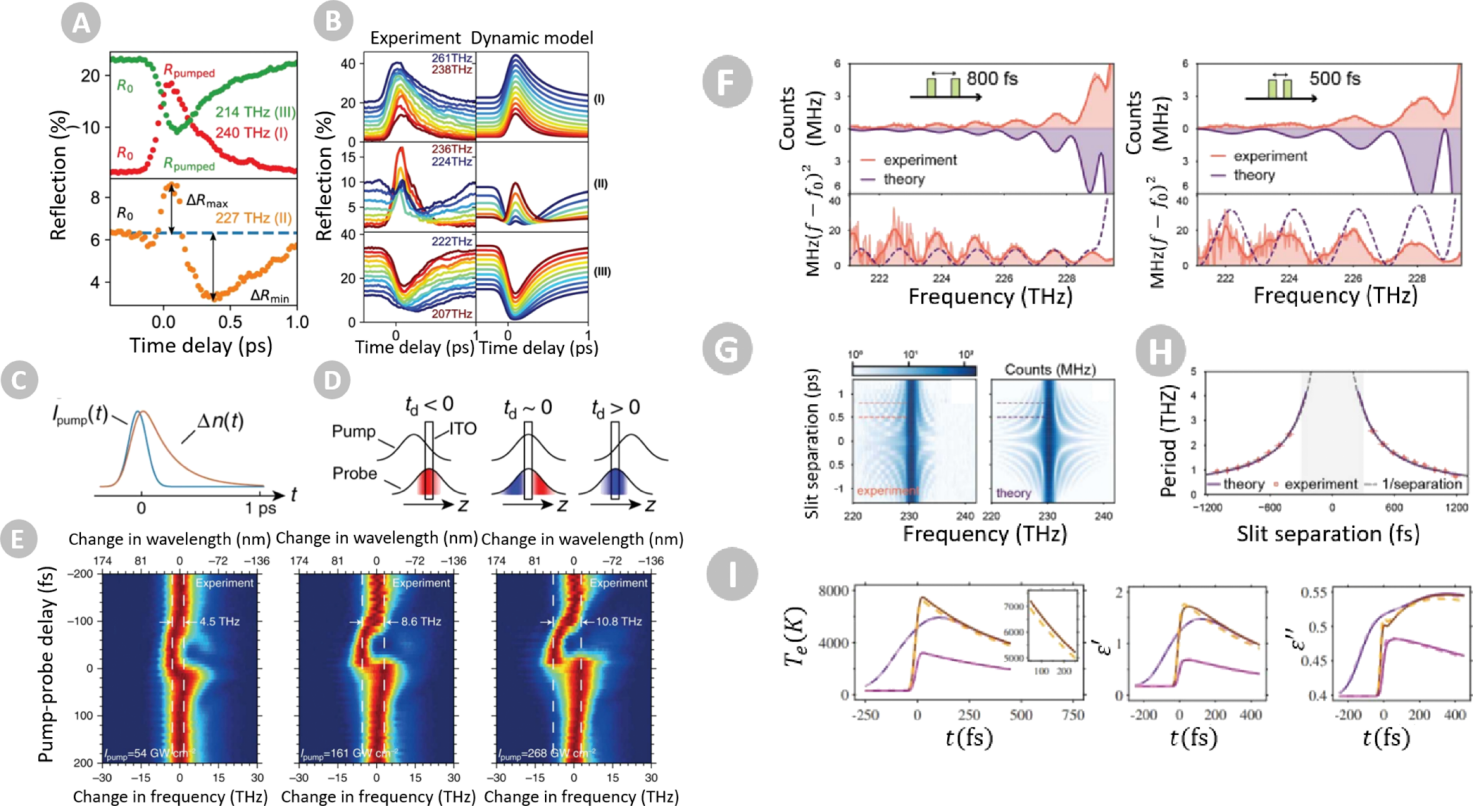
(a, b) All-optical switching of an ENZ plasmon resonance in ITO, showing subpicosecond amplitude modulation of a reflected signal produced by an ultrafast shift in its plasma frequency. Reproduced with permission from ref (192). Copyright 2021 Nature. (c, d) Illustration of a broadband frequency translation through time refraction in an ENZ material, and (e) its measurement in ITO for increasing pump intensities.^177^ (f) Experimental measurement (red) and theoretical prediction (blue) of double-slit time diffraction, produced by shining two pump pulses separated by a delay of (left) 800 fs and (right) 500 fs, resulting in different diffraction fringes. Reproduced with permission from ref (178). Copyright 2023 Nature. (g) Experimental (left) and theoretical (right) field intensities from a double-slit time diffraction as a function of frequency and slit separation, quantitatively compared in panel (h). (i) Time-dependence of (left) the electron temperature, (middle) real and (right) imaginary parts of the ITO permittivity under optical pumping via (purple) a 220 fs pulse at an intensity of 22 GW/cm^2^, (orange) a 20 fs pulse at 161 GW/cm^2^ and (magenta) 30 fs at 22 GW/cm^2^, clearly predicting femtosecond-scale responses in ITO. Reproduced with permission from ref (190). Copyright 2023 APS.

Following the pioneering demonstration of the unmatched strength of their nonlinearities,^91,95^ ENZ media, especially ITO, have gained a spotlight in the quest to implement giant, ultrafast permittivity modulations at near-optical frequencies. Early explorations led to the observation of giant sub-ps amplitude modulation via ultrafast shifts of the ENZ frequency of ITO, both by exploiting the coupling to leaky modes^186^ and to evanescent ones^124,187^ (Figure 5a,b).

Currently, efforts are shifting toward using ENZ media as efficient platforms for time-varying wave physics at near-optical frequencies to establish new paradigms for spectral control. Crucially, this endeavor necessarily entails probing the intrinsic modulation speeds available in these materials. A pioneering study demonstrated the temporal analogue of refraction at the interface between two media, a process whereby a change in the refractive index of one of them induces a change in the frequency of light while conserving its momentum.^177^ By inducing a large change in the optical properties of a 620 nm ITO film, an extremely broadband and controllable frequency translation of up to 14.9 THz was observed in a copropagating probe (Figure 5c–e). At the quantum level, time-varying ITO in combination with gold nanoantennas has been exploited to spontaneously generate photon pairs from the quantum vacuum.^188^ More recently, the temporal analogue of Young’s double slit diffraction experiment in photonics was reported^178^ (Figure 5f–h) more than 50 years after its prediction.^189^ Most remarkably, this experiment revealed the unexpectedly fast nonlinear response of ITO,^178^ estimating rise times of less than 10 fs, which sparked ongoing theoretical investigations on the nature of such unprecedented response times and the search for new materials exhibiting ultrafast responses of similar time scales. These studies are currently unveiling the key role of momentum conservation in the electron–phonon interaction in such low-electron-density Drude materials, which leads them to support 8-fold electron temperatures compared to standard plasmonic materials under analogous illumination conditions (Figure 5i).^190,191^

Advances in the quest to achieve single-cycle modulation time scales at near-optical frequencies are further stimulating new theoretical developments toward the efficient modeling of time-varying media. Time-varying effects in subwavelength nanostructures introduce unique challenges,^193^ as the spatial and temporal scales involved can span several orders of magnitude, and their resolution needs to be comparable in finite-differencing schemes to ensure numerical stability. In order to overcome adiabatic approximations,^177,192^ more efficient scattering paradigms and techniques are being steadily developed, including novel approaches to deal with the interplay between temporal dependence and frequency dispersion.^194,195^ At the heart of this, however, are fundamental theoretical challenges concerning boundary conditions and conservation laws for electromagnetic fields at temporal inhomogeneities, a field of intense ongoing investigation for basic electromagnetics research.^181,196,197^

In turn, all these advances in the ultrafast, giant temporal modulation of ENZ media promise a plethora of exciting ideas to be tested in time-varying photonic platforms. Importantly, the possibility of strong modulations at single-cycle time scales may lead to the realization of temporal photonic crystals.^198^ Furthermore, other exotic ideas may soon be realized, such as implementing spatiotemporal modulations^199^ and non-parametric gain,^200,201^ chiral pulse amplification,^202^ or Floquet topological modes.^203^ Further possibilities include enhanced emission and mirrorless lasing,^198^ subdiffractional-mode excitation on non-structured surfaces,^204^ the spontaneous generation of polariton pairs from the quantum vacuum through the dynamic Casimir effect,^205−207^ the control over all entanglement degrees of freedom of single photons,^208^ and the enhancement and tailoring of spontaneous emission of free electrons.^209^

Finally, in the context of the topic treated in this section, it is worth closing the circle by making a connection with a topic treated in the section Engineering of Light and Thermal Emission in NZI Media. In fact, new opportunities for the engineering of thermal emission are opened when NZI materials are modulated in time.^210^ Time-modulation of the refractive index breaks key assumptions in the usual form of the fluctuation dissipation theorem^211^ and Kirchhoff’s law,^212^ which form the basis of thermal emitters. Therefore, while thermal fluctuating currents are typically uncorrelated in frequency and space for conventional thermal emitters, time modulation leads to secondary currents that are correlated in frequency and space, opening the door to thermal emission with enhanced coherence and nontrivial photon correlations.^213^ Furthermore, energy can be either pumped into a material or retracted from it as it is modulated in time, enabling “active” thermal emitters radiating outside the blackbody spectrum,^213^ and acting as heat engines.^214^ Thermal emission from NZI bodies is particularly sensitive to time modulation. For example, since the near-field of a fluctuating current scale as *E*_NF_ ∼ 1/(4πε*r*^3^), ENZ bodies support very strong thermal fields within them. Temporal modulation is capable of releasing these fields, forming the dual of a spatial grating, which consists of a narrowband peak fixed at a given frequency, but whose radiation scans all wave-vectors, from near to far fields.^213^

## Conclusions

We highlighted the tremendous activity of a vibrant research community demonstrating the capabilities of NZI systems and HMM metamaterials to manipulate light-matter interactions in both the frequency and time domain. Engineering of ε(*r⃗*,*t*), and consequently *n*(*r⃗*,*t*), around their near-zero value broadens the horizons in several areas, including light and thermal emission, nonlinear optics, and all-optical switching, as well as sensing and quantum applications. NZI materials are also a promising platform for exploring the emerging field of time-varying photonics.

Nevertheless, while providing several unique benefits and demonstrating the aforementioned breakthroughs, NZI and HMM research field still face challenges that need to be overcome such as intrinsic ohmic losses of metallic inclusions, reducing its applicability, for instance in sensing. Routes to boost performance of HMM biosensors include the use of nanocavities in multilayer metamaterials (to increase the sensitivity through enhanced electromagnetic field–analyte interactions) or MO effects (to improve resolution). Based on recent developments mentioned in this Perspective, we may foresee the use of plasmonic nanocavities in MO multilayer HMM for future ultrasensitive and ultrahigh resolution biosensors. Moreover, optical forces due to the highly confined electromagnetic fields into deep subwavelength plasmonic nanocavities can provide a way to beat the need to use binding tethers or labeling (e.g., fluorophores),^215−217^ improving device recyclability in future developments. Further exciting possibilities might also come from the field of ultrafast magnetism, as recently it has been shown the potential of using ENZ materials for manipulating functional properties of solids.^218^

In addition, as we discussed in the last section, ENZ media are also being employed as one of the main platforms for exploring photonics in time-varying media. The underlying reason is their unique capability to provide ultrafast and strong changes of their optical response in the near infrared through nonlinear effects rooted in nonequilibrium electron dynamics. Thus, ENZ materials provide a ground breaking platform for exploring new regimes of light-matter interactions. Amidst the quest for translating the growing, rich phenomenology of time-varying media toward the visible range, mounting experimental and theoretical evidence points at the prime role that ENZ media will play over the coming years, in turn feeding back new insights into their nontrivial nonequilibrium dynamics.

Finally, ENZ conditions provide several benefits to nonlinear optics thanks to the versatile control over material dispersion. Nevertheless, such a condition implies optical loss and moderate enhancement. We see potential benefits in areas where control over high irradiances is needed or in scenarios where narrow operating bandwidths are utilized, as well as in the use of weakly resonant structures, such as plasmonic antennas, to provide a middle ground wherein the operational spectral bandwidth can remain reasonably broad (∼100 nm) while gaining additional improvement to the nonlinearity. To conclude, the fundamental question facing the community is whether the benefit of ENZ condition and hyperbolic dispersion can overcome their limitations to provide relevant applications. Nevertheless, we should look at the future with optimism, as the current advances in the field, in particular in engineering HMM structures for improving sensing capabilities or exploiting ohmic losses in the context of light and thermal emission modulation, as well as recent experimental breakthroughs in the field of time-varying media, make us confident that this field is thriving and will be full of surprises in the upcoming years.

## References

[ref1] Saleh B. E. A.; Teich M. C. Fundamentals of Photonics; Wiley-Interscience: Hoboken, NJ, 2001.

[ref2] Maier S. A. Plasmonics: Fundamentals and Applications; Springer US, 2007. 10.1007/0-387-37825-1.

[ref3] Photonic Crystals: Molding the Flow of Light, 2nd ed.; Joannopoulos J. D., Ed.; Princeton University Press, 2008.

[ref4] Novotny L.; Hecht B. Principles of Nano-Optics; Cambridge University Press, 2012. 10.1017/CBO9780511794193.

[ref5] Yablonovitch E. Inhibited Spontaneous Emission in Solid-State Physics and Electronics. Phys. Rev. Lett. 1987, 58 (20), 2059–2062. 10.1103/PhysRevLett.58.2059. 10034639

[ref6] John S. Strong Localization of Photons in Certain Disordered Dielectric Superlattices. Phys. Rev. Lett. 1987, 58 (23), 2486–2489. 10.1103/PhysRevLett.58.2486. 10034761

[ref7] Engheta N.; Ziolkowski R. W. Metamaterials: Physics and Engineering Explorations; Wiley-Interscience: Hoboken, N.J, 2010.

[ref8] Liberal I.; Engheta N. Near-Zero Refractive Index Photonics. Nature Photon 2017, 11 (3), 149–158. 10.1038/nphoton.2017.13.

[ref9] Kinsey N.; DeVault C.; Boltasseva A.; Shalaev V. M. Near-Zero-Index Materials for Photonics. Nat. Rev. Mater. 2019, 4 (12), 742–760. 10.1038/s41578-019-0133-0.

[ref10] Vulis D. I.; Reshef O.; Camayd-Muñoz P.; Mazur E. Manipulating the Flow of Light Using Dirac-Cone Zero-Index Metamaterials. Rep. Prog. Phys. 2019, 82 (1), 012001 10.1088/1361-6633/aad3e5. 30015328

[ref11] Poddubny A.; Iorsh I.; Belov P.; Kivshar Y. Hyperbolic Metamaterials. Nature Photon 2013, 7 (12), 948–957. 10.1038/nphoton.2013.243.

[ref12] Argyropoulos C.; Estakhri N. M.; Monticone F.; Alù A. Negative Refraction, Gain and Nonlinear Effects in Hyperbolic Metamaterials. Opt. Express 2013, 21 (12), 15037 10.1364/OE.21.015037. 23787691

[ref13] Ferrari L.; Wu C.; Lepage D.; Zhang X.; Liu Z. Hyperbolic Metamaterials and Their Applications. Progress in Quantum Electronics 2015, 40, 1–40. 10.1016/j.pquantelec.2014.10.001.

[ref14] Huo P.; Zhang S.; Liang Y.; Lu Y.; Xu T. Hyperbolic Metamaterials and Metasurfaces: Fundamentals and Applications. Adv. Optical Mater. 2019, 7 (14), 1801616 10.1002/adom.201801616.

[ref15] Takayama O.; Lavrinenko A. V. Optics with Hyperbolic Materials [Invited]. J. Opt. Soc. Am. B 2019, 36 (8), F38 10.1364/JOSAB.36.000F38.

[ref16] Palermo G.; Sreekanth K. V.; Strangi G. Hyperbolic Dispersion Metamaterials and Metasurfaces. EPJ. Appl. Metamat. 2020, 7, 11 10.1051/epjam/2020015.

[ref17] Guo Z.; Jiang H.; Chen H. Hyperbolic Metamaterials: From Dispersion Manipulation to Applications. J. Appl. Phys. 2020, 127 (7), 071101 10.1063/1.5128679.

[ref18] Hecht E. Optics, 5th ed.; Pearson Education, Inc: Boston, 2017.

[ref19] Silveirinha M.; Engheta N. Tunneling of Electromagnetic Energy through Subwavelength Channels and Bends Using ε -Near-Zero Materials. Phys. Rev. Lett. 2006, 97 (15), 157403 10.1103/PhysRevLett.97.157403. 17155357

[ref20] Lobet M.; Liberal I.; Knall E. N.; Alam M. Z.; Reshef O.; Boyd R. W.; Engheta N.; Mazur E. Fundamental Radiative Processes in Near-Zero-Index Media of Various Dimensionalities. ACS Photonics 2020, 7 (8), 1965–1970. 10.1021/acsphotonics.0c00782.

[ref21] Hao J.; Yan W.; Qiu M. Super-Reflection and Cloaking Based on Zero Index Metamaterial. Appl. Phys. Lett. 2010, 96 (10), 101109 10.1063/1.3359428.

[ref22] Huang X.; Lai Y.; Hang Z. H.; Zheng H.; Chan C. T. Dirac Cones Induced by Accidental Degeneracy in Photonic Crystals and Zero-Refractive-Index Materials. Nat. Mater. 2011, 10 (8), 582–586. 10.1038/nmat3030. 21623377

[ref23] Ploss D.; Kriesch A.; Etrich C.; Engheta N.; Peschel U. Young’s Double-Slit, Invisible Objects and the Role of Noise in an Optical Epsilon-near-Zero Experiment. ACS Photonics 2017, 4 (10), 2566–2572. 10.1021/acsphotonics.7b00861.

[ref24] Ziolkowski R. Metamaterial-Based Source and Scattering Enhancements: From Microwave to Optical Frequencies. Opto-Electronics Review 2006, 14 (3), 167–177. 10.2478/s11772-006-0022-0.

[ref25] Cai W.; Šalaev V. M. Optical Metamaterials: Fundamentals and Applications; Springer: New York, NY, Heidelberg, 2010.

[ref26] Narimanov E. E.; Kildishev A. V. Naturally Hyperbolic. Nature Photon 2015, 9 (4), 214–216. 10.1038/nphoton.2015.56.

[ref27] Yoxall E.; Schnell M.; Nikitin A. Y.; Txoperena O.; Woessner A.; Lundeberg M. B.; Casanova F.; Hueso L. E.; Koppens F. H. L.; Hillenbrand R. Direct Observation of Ultraslow Hyperbolic Polariton Propagation with Negative Phase Velocity. Nature Photon 2015, 9 (10), 674–678. 10.1038/nphoton.2015.166.

[ref28] Li P.; Dolado I.; Alfaro-Mozaz F. J.; Nikitin A. Yu.; Casanova F.; Hueso L. E.; Vélez S.; Hillenbrand R. Optical Nanoimaging of Hyperbolic Surface Polaritons at the Edges of van Der Waals Materials. Nano Lett. 2017, 17 (1), 228–235. 10.1021/acs.nanolett.6b03920. 27966994

[ref29] Govyadinov A. A.; Konečná A.; Chuvilin A.; Vélez S.; Dolado I.; Nikitin A. Y.; Lopatin S.; Casanova F.; Hueso L. E.; Aizpurua J.; Hillenbrand R. Probing Low-Energy Hyperbolic Polaritons in van Der Waals Crystals with an Electron Microscope. Nat. Commun. 2017, 8 (1), 95 10.1038/s41467-017-00056-y. 28733660 PMC5522439

[ref30] Dai S.; Tymchenko M.; Yang Y.; Ma Q.; Pita-Vidal M.; Watanabe K.; Taniguchi T.; Jarillo-Herrero P.; Fogler M. M.; Alù A.; Basov D. N. Manipulation and Steering of Hyperbolic Surface Polaritons in Hexagonal Boron Nitride. Adv. Mater. 2018, 30 (16), 1706358 10.1002/adma.201706358. 29532960

[ref31] Dai S.; Quan J.; Hu G.; Qiu C.-W.; Tao T. H.; Li X.; Alù A. Hyperbolic Phonon Polaritons in Suspended Hexagonal Boron Nitride. Nano Lett. 2019, 19 (2), 1009–1014. 10.1021/acs.nanolett.8b04242. 30550296

[ref32] Ma W.; Hu G.; Hu D.; Chen R.; Sun T.; Zhang X.; Dai Q.; Zeng Y.; Alù A.; Qiu C.-W.; Li P. Ghost Hyperbolic Surface Polaritons in Bulk Anisotropic Crystals. Nature 2021, 596 (7872), 362–366. 10.1038/s41586-021-03755-1. 34408329

[ref33] Passler N. C.; Ni X.; Hu G.; Matson J. R.; Carini G.; Wolf M.; Schubert M.; Alù A.; Caldwell J. D.; Folland T. G.; Paarmann A. Hyperbolic Shear Polaritons in Low-Symmetry Crystals. Nature 2022, 602 (7898), 595–600. 10.1038/s41586-021-04328-y. 35197618 PMC8866127

[ref34] Liberal I.; Engheta N. Zero-Index Structures as an Alternative Platform for Quantum Optics. Proc. Natl. Acad. Sci. U.S.A. 2017, 114 (5), 822–827. 10.1073/pnas.1611924114. 28096367 PMC5293079

[ref35] Liberal I.; Engheta N. Nonradiating and Radiating Modes Excited by Quantum Emitters in Open Epsilon-near-Zero Cavities. Sci. Adv. 2016, 2 (10), e1600987 10.1126/sciadv.1600987. 27819047 PMC5088639

[ref36] Vesseur E. J. R.; Coenen T.; Caglayan H.; Engheta N.; Polman A. Experimental Verification of n = 0 Structures for Visible Light. Phys. Rev. Lett. 2013, 110 (1), 013902 10.1103/PhysRevLett.110.013902. 23383791

[ref37] So J.-K.; Yuan G. H.; Soci C.; Zheludev N. I. Enhancement of Luminescence of Quantum Emitters in Epsilon-near-Zero Waveguides. Appl. Phys. Lett. 2020, 117 (18), 181104 10.1063/5.0018488.

[ref38] Contractor R.; Noh W.; Redjem W.; Qarony W.; Martin E.; Dhuey S.; Schwartzberg A.; Kanté B. Scalable Single-Mode Surface-Emitting Laser via Open-Dirac Singularities. Nature 2022, 608 (7924), 692–698. 10.1038/s41586-022-05021-4. 35768016

[ref39] Lončar M.; Yoshie T.; Scherer A.; Gogna P.; Qiu Y. Low-Threshold Photonic Crystal Laser. Appl. Phys. Lett. 2002, 81 (15), 2680–2682. 10.1063/1.1511538.

[ref40] Bohnet J. G.; Chen Z.; Weiner J. M.; Meiser D.; Holland M. J.; Thompson J. K. A Steady-State Superradiant Laser with Less than One Intracavity Photon. Nature 2012, 484 (7392), 78–81. 10.1038/nature10920. 22481360

[ref41] Liberal I.; Ziolkowski R. W. Nonperturbative Decay Dynamics in Metamaterial Waveguides. Appl. Phys. Lett. 2021, 118 (11), 111103 10.1063/5.0044103.

[ref42] Bello M.; Platero G.; González-Tudela A. Spin Many-Body Phases in Standard- and Topological-Waveguide QED Simulators. PRX Quantum 2022, 3 (1), 010336 10.1103/PRXQuantum.3.010336.

[ref43] Fleury R.; Alù A. Enhanced Superradiance in Epsilon-near-Zero Plasmonic Channels. Phys. Rev. B 2013, 87 (20), 201101 10.1103/PhysRevB.87.201101.

[ref44] Mello O.; Li Y.; Camayd-Muñoz S. A.; DeVault C.; Lobet M.; Tang H.; Lonçar M.; Mazur E. Extended Many-Body Superradiance in Diamond Epsilon near-Zero Metamaterials. Appl. Phys. Lett. 2022, 120 (6), 061105 10.1063/5.0062869.

[ref45] Sokhoyan R.; Atwater H. A. Quantum Optical Properties of a Dipole Emitter Coupled to an ε-near-Zero Nanoscale Waveguide. Opt. Express 2013, 21 (26), 32279 10.1364/OE.21.032279. 24514821

[ref46] Özgün E.; Ozbay E.; Caglayan H. Tunable Zero-Index Photonic Crystal Waveguide for Two-Qubit Entanglement Detection. ACS Photonics 2016, 3 (11), 2129–2133. 10.1021/acsphotonics.6b00576.

[ref47] Liberal I.; Engheta N. Multiqubit Subradiant States in N -Port Waveguide Devices: ε-and-μ-near-Zero Hubs and Nonreciprocal Circulators. Phys. Rev. A 2018, 97 (2), 022309 10.1103/PhysRevA.97.022309.

[ref48] Li Y.; Nemilentsau A.; Argyropoulos C. Resonance Energy Transfer and Quantum Entanglement Mediated by Epsilon-near-Zero and Other Plasmonic Waveguide Systems. Nanoscale 2019, 11 (31), 14635–14647. 10.1039/C9NR05083C. 31343051

[ref49] Issah I.; Habib M.; Caglayan H. Long-Range Qubit Entanglement via Rolled-up Zero-Index Waveguide. Nanophotonics 2021, 10 (18), 4579–4589. 10.1515/nanoph-2021-0453.

[ref50] Aspect A.; Grangier P.; Roger G. Experimental Realization of Einstein-Podolsky-Rosen-Bohm *Gedankenexperiment* : A New Violation of Bell’s Inequalities. Phys. Rev. Lett. 1982, 49 (2), 91–94. 10.1103/PhysRevLett.49.91.

[ref51] Aspect A.; Dalibard J.; Roger G. Experimental Test of Bell’s Inequalities Using Time- Varying Analyzers. Phys. Rev. Lett. 1982, 49 (25), 1804–1807. 10.1103/PhysRevLett.49.1804.

[ref52] Martín-Cano D.; González-Tudela A.; Martín-Moreno L.; García-Vidal F. J.; Tejedor C.; Moreno E. Dissipation-Driven Generation of Two-Qubit Entanglement Mediated by Plasmonic Waveguides. Phys. Rev. B 2011, 84 (23), 235306 10.1103/PhysRevB.84.235306. 21405211

[ref53] Bittencourt V. A. S. V.; Liberal I.; Viola Kusminskiy S. Optomagnonics in Dispersive Media: Magnon-Photon Coupling Enhancement at the Epsilon-near-Zero Frequency. Phys. Rev. Lett. 2022, 128 (18), 183603 10.1103/PhysRevLett.128.183603. 35594084

[ref54] Bittencourt V. A. S. V.; Liberal I.; Viola Kusminskiy S. Light Propagation and Magnon-Photon Coupling in Optically Dispersive Magnetic Media. Phys. Rev. B 2022, 105 (1), 014409 10.1103/PhysRevB.105.014409. 35594084

[ref55] Einstein A. Zur Quantentheorie Der Strahlung. Mitteilungen Phys. Ges. Zür 1916, 16, 47–62.

[ref56] Einstein A. Zur Quantentheorie Der Strahlung. Phys. Z. 1917, 18, 121–128.

[ref57] Lobet M.; Liberal I.; Vertchenko L.; Lavrinenko A. V.; Engheta N.; Mazur E. Momentum Considerations inside Near-Zero Index Materials. Light Sci. Appl. 2022, 11 (1), 110 10.1038/s41377-022-00790-z. 35468887 PMC9039083

[ref58] Minkowski H. Die Grundgleichungen für die elektromagnetischen Vorgänge in bewegten Körpern. Math. Ann. 1910, 68 (4), 472–525. 10.1007/BF01455871.

[ref59] Abraham M. Zur Elektrodynamik Bewegter Körper. Rendiconti Circolo Mater. Palermo 1909, 28, 1 10.1007/BF03018208.

[ref60] Abraham M. Sull’elettrodinamica Di Minkowski. Rendiconti Circolo Mater. Palermo 1910, 30, 33–46. 10.1007/BF03014862.

[ref61] Barnett S. M. Resolution of the Abraham-Minkowski Dilemma. Phys. Rev. Lett. 2010, 104 (7), 070401 10.1103/PhysRevLett.104.070401. 20366861

[ref62] Leonhardt U. Momentum in an Uncertain Light. Nature 2006, 444 (7121), 823–824. 10.1038/444823a. 17167461

[ref63] Kinsey N. Developing Momentum in Vanishing Index Photonics. Light Sci. Appl. 2022, 11 (1), 148 10.1038/s41377-022-00846-0. 35595801 PMC9123175

[ref64] Fan S. Thermal Photonics and Energy Applications. Joule 2017, 1 (2), 264–273. 10.1016/j.joule.2017.07.012.

[ref65] Li W.; Fan S. Nanophotonic Control of Thermal Radiation for Energy Applications [Invited]. Opt. Express 2018, 26 (12), 15995 10.1364/OE.26.015995. 30114851

[ref66] Picardi M. F.; Nimje K. N.; Papadakis G. T. Dynamic Modulation of Thermal Emission—A Tutorial. J. Appl. Phys. 2023, 133 (11), 111101 10.1063/5.0134951.

[ref67] Liberal I.; Engheta N. Manipulating Thermal Emission with Spatially Static Fluctuating Fields in Arbitrarily Shaped Epsilon-near-Zero Bodies. Proc. Natl. Acad. Sci. U.S.A. 2018, 115 (12), 2878–2883. 10.1073/pnas.1718264115. 29507219 PMC5866572

[ref68] Molesky S.; Dewalt C. J.; Jacob Z. High Temperature Epsilon-near-Zero and Epsilon-near-Pole Metamaterial Emitters for Thermophotovoltaics. Opt. Express 2013, 21 (S1), A96 10.1364/OE.21.000A96. 23389280

[ref69] Dyachenko P. N.; Molesky S.; Petrov A. Y.; Störmer M.; Krekeler T.; Lang S.; Ritter M.; Jacob Z.; Eich M. Controlling Thermal Emission with Refractory Epsilon-near-Zero Metamaterials via Topological Transitions. Nat. Commun. 2016, 7 (1), 11809 10.1038/ncomms11809. 27263653 PMC4897762

[ref70] Sievenpiper D.; Zhang L.; Broas R. F. J.; Alexopolous N. G.; Yablonovitch E. High-Impedance Electromagnetic Surfaces with a Forbidden Frequency Band. IEEE Trans. Microwave Theory Techn. 1999, 47 (11), 2059–2074. 10.1109/22.798001.

[ref71] Feresidis A. P.; Goussetis G.; Wang S.; Vardaxoglou J. C. Artificial Magnetic Conductor Surfaces and Their Application to Low-Profile High-Gain Planar Antennas. IEEE Trans. Antennas Propagat. 2005, 53 (1), 209–215. 10.1109/TAP.2004.840528.

[ref72] Navajas D.; Pérez-Escudero J. M.; Liberal I. Spectrally Stable Thermal Emitters Enabled by Material-Based High-Impedance Surfaces. Nanoscale Adv. 2023, 5 (3), 650–658. 10.1039/D2NA00633B. 36756519 PMC9890674

[ref73] Pérez-Escudero J. M.; Buldain I.; Beruete M.; Goicoechea J.; Liberal I. Silicon Carbide as a Material-Based High-Impedance Surface for Enhanced Absorption within Ultra-Thin Metallic Films. Opt. Express 2020, 28 (21), 31624 10.1364/OE.402397. 33115132

[ref74] Kim B. G.; Garmire E.; Hummel S. G.; Dapkus P. D. Nonlinear Bragg Reflector Based on Saturable Absorption. Appl. Phys. Lett. 1989, 54 (12), 1095–1097. 10.1063/1.100768.

[ref75] Keller U.; Weingarten K. J.; Kartner F. X.; Kopf D.; Braun B.; Jung I. D.; Fluck R.; Honninger C.; Matuschek N.; Aus Der Au J. Semiconductor Saturable Absorber Mirrors (SESAM’s) for Femtosecond to Nanosecond Pulse Generation in Solid-State Lasers. IEEE J. Select. Topics Quantum Electron. 1996, 2 (3), 435–453. 10.1109/2944.571743.

[ref76] Jung I. D.; Kärtner F. X.; Matuschek N.; Sutter D. H.; Morier-Genoud F.; Shi Z.; Scheuer V.; Tilsch M.; Tschudi T.; Keller U. Semiconductor Saturable Absorber Mirrors Supporting Sub-10-Fs Pulses. Applied Physics B: Lasers and Optics 1997, 65 (2), 137–150. 10.1007/s003400050259.

[ref77] Wu S.-T.; Wu C.-S.; Lim K.-C.; Hsu T.-Y. Patent Application No. 08/552412, June 2, 1998.

[ref78] Martinsen G.; Havig P.; Dykes J.; Kuyk T.; McLin L. In Night Vision Goggles, Laser Eye Protection, and Cockpit Displays; Brown R. W., Reese C. E., Marasco P. L., Harding T. H., Eds.; Defense and Security Symposium, Orlando, Florida, U.S.A., 2007; p 65570V. 10.1117/12.720864.

[ref79] Khurgin J. Electro-optical Switching and Bistability in Coupled Quantum Wells. Appl. Phys. Lett. 1989, 54 (25), 2589–2591. 10.1063/1.101058.

[ref80] Boyd R. W. Nonlinear Opt., 3rd ed.; Elsevier, Academic Press: Amsterdam; Heidelberg, 2008.

[ref81] Miller D. A. B.; Feuer M. D.; Chang T. Y.; Shunk S. C.; Henry J. E.; Burrows D. J.; Chemla D. S. Field-Effect Transistor Self-Electrooptic Effect Device: Integrated Photodiode, Quantum Well Modulator and Transistor. IEEE Photon. Technol. Lett. 1989, 1 (3), 62–64. 10.1109/68.87897.

[ref82] Miller D. A. B. The Role of Optics in Computing. Nature Photon 2010, 4 (7), 406–406. 10.1038/nphoton.2010.163.

[ref83] Ambs P. Optical Computing: A 60-Year Adventure. Advances in Optical Technologies 2010, 2010, 1–15. 10.1155/2010/372652.

[ref84] Hau L. V.; Harris S. E.; Dutton Z.; Behroozi C. H. Light Speed Reduction to 17 Metres per Second in an Ultracold Atomic Gas. Nature 1999, 397 (6720), 594–598. 10.1038/17561.

[ref85] Krauss T. F. Why Do We Need Slow Light?. Nature Photon 2008, 2 (8), 448–450. 10.1038/nphoton.2008.139.

[ref86] Khurgin J. B. Slow Light in Various Media: A Tutorial. Adv. Opt. Photon. 2010, 2 (3), 287 10.1364/AOP.2.000287.

[ref87] Boyd R. W. Material Slow Light and Structural Slow Light: Similarities and Differences for Nonlinear Optics [Invited]. J. Opt. Soc. Am. B 2011, 28 (12), A38 10.1364/JOSAB.28.000A38.

[ref88] Khurgin J. B.; Clerici M.; Bruno V.; Caspani L.; DeVault C.; Kim J.; Shaltout A.; Boltasseva A.; Shalaev V. M.; Ferrera M.; Faccio D.; Kinsey N. Adiabatic Frequency Shifting in Epsilon-near-Zero Materials: The Role of Group Velocity. Optica 2020, 7 (3), 226 10.1364/OPTICA.374788.

[ref89] Khurgin J. B.; Clerici M.; Kinsey N. Fast and Slow Nonlinearities in Epsilon-Near-Zero Materials. Laser & Photonics Reviews 2021, 15 (2), 2000291 10.1002/lpor.202000291.

[ref90] Kinsey N.; DeVault C.; Boltasseva A.; Shalaev V. M. V. M. Near-Zero-Index Materials for Photonics. Nature Reviews Materials 2019, 4 (12), 742–760. 10.1038/s41578-019-0133-0.

[ref91] Reshef O.; De Leon I.; Alam M. Z.; Boyd R. W. Nonlinear Optical Effects in Epsilon-near-Zero Media. Nat. Rev. Mater. 2019, 4 (8), 535–551. 10.1038/s41578-019-0120-5.

[ref92] Fruhling C.; Ozlu M. G.; Saha S.; Boltasseva A.; Shalaev V. M. Understanding All-Optical Switching at the Epsilon-near-Zero Point: A Tutorial Review. Appl. Phys. B: Laser Opt. 2022, 128 (2), 34 10.1007/s00340-022-07756-4.

[ref93] Kinsey N.; DeVault C.; Kim J.; Ferrera M.; Shalaev V. M.; Boltasseva A. Epsilon-near-Zero Al-Doped ZnO for Ultrafast Switching at Telecom Wavelengths. Optica 2015, 2 (7), 616–622. 10.1364/OPTICA.2.000616.

[ref94] Caspani L.; Kaipurath R. P. M.; Clerici M.; Ferrera M.; Roger T.; Kim J.; Kinsey N.; Pietrzyk M.; Di Falco A.; Shalaev V. M.; Boltasseva A.; Faccio D. Enhanced Nonlinear Refractive Index in Epsilon-near-Zero Materials. Phys. Rev. Lett. 2016, 116 (23), 239901 10.1103/PhysRevLett.116.233901. 27341234

[ref95] Alam M. Z.; De Leon I.; Boyd R. W. Large Optical Nonlinearity of Indium Tin Oxide in Its Epsilon-near-Zero Region. Science 2016, 352 (6287), 795–797. 10.1126/science.aae0330. 27127238

[ref96] Rashed A. R.; Yildiz B. C.; Ayyagari S. R.; Caglayan H. Hot Electron Dynamics in Ultrafast Multilayer Epsilon-near-Zero Metamaterials. Phys. Rev. B 2020, 101 (16), 165301 10.1103/PhysRevB.101.165301.

[ref97] Kuttruff J.; Garoli D.; Allerbeck J.; Krahne R.; De Luca A.; Brida D.; Caligiuri V.; Maccaferri N. Ultrafast All-Optical Switching Enabled by Epsilon-near-Zero-Tailored Absorption in Metal-Insulator Nanocavities. Commun. Phys. 2020, 3 (1), 114 10.1038/s42005-020-0379-2.

[ref98] Boyd R. Nonlinear Opt., 3rd ed.; Elsevier: Burlington, MA, 2008.

[ref99] Dinu M.; Quochi F.; Garcia H. Third-Order Nonlinearities in Silicon at Telecom Wavelengths. Appl. Phys. Lett. 2003, 82 (18), 2954–2956. 10.1063/1.1571665.

[ref100] Hurlbut W. C.; Lee Y.-S.; Vodopyanov K. L.; Kuo P. S.; Fejer M. M. Multiphoton Absorption and Nonlinear Refraction of GaAs in the Mid-Infrared. Opt. Lett. 2007, 32 (6), 668 10.1364/OL.32.000668. 17308596

[ref101] Benis S.; Munera N.; Acuña R.; Hagan D. J.; Van Stryland E. W. Nonlinear Fresnel Coefficients Due to Giant Ultrafast Nonlinearities in Indium Tin Oxide (Conference Presentation). In Ultrafast Phenomena and Nanophotonics XXIII; Betz M., Elezzabi A. Y., Eds.; SPIE, 2019; Vol. 10916, p 35. 10.1117/12.2510690.

[ref102] Ball A.; Secondo R.; Diroll B. T.; Fomra D.; Ding K.; Avrutin V.; Özgür D. C.; Kinsey N. Gallium-Doped Zinc Oxide: Nonlinear Reflection and Transmission Measurements and Modeling in the ENZ Region. Journal of Physics: Photonics 2023, 5 (2), 024001 10.1088/2515-7647/acbdd7.

[ref103] Alam M. Z.; Schulz S. A.; Upham J.; De Leon I.; Boyd R. W. Large Optical Nonlinearity of Nanoantennas Coupled to an Epsilon-near-Zero Material. Nat. Photonics 2018, 12 (2), 79–83. 10.1038/s41566-017-0089-9.

[ref104] Benis S.; Munera N.; Faryadras S.; Van Stryland E. W.; Hagan D. J. Extremely Large Nondegenerate Nonlinear Index and Phase Shift in Epsilon-near-Zero Materials [Invited]. Optical Materials Express 2022, 12 (10), 3856 10.1364/OME.464846.

[ref105] Benjamin S. D.; Loka H. S.; Othonos A.; Smith P. W. E. Ultrafast Dynamics of Nonlinear Absorption in Low-temperature-grown GaAs. Appl. Phys. Lett. 1996, 68 (18), 2544–2546. 10.1063/1.116178.

[ref106] Vermeulen N.; Espinosa D.; Ball A.; Ballato J.; Boucaud P.; Boudebs G.; Campos C. L A V; Dragic P.; Gomes A. S L; Huttunen M. J; Kinsey N.; Mildren R.; Neshev D.; Padilha L. A; Pu M.; Secondo R.; Tokunaga E.; Turchinovich D.; Yan J.; Yvind K.; Dolgaleva K.; Van Stryland E. W Post-2000 Nonlinear Optical Materials and Measurements: Data Tables and Best Practices. Journal of Physics: Photonics 2023, 5 (3), 035001 10.1088/2515-7647/ac9e2f.

[ref107] Kinsey N.; Khurgin J. Nonlinear Epsilon-near-Zero Materials Explained: Opinion. Optical Materials Express 2019, 9 (7), 2793 10.1364/OME.9.002793.

[ref108] Khurgin J. B. How to Deal with the Loss in Plasmonics and Metamaterials. Nat. Nanotechnol. 2015, 10 (1), 2–6. 10.1038/nnano.2014.310. 25559961

[ref109] Khurgin J. B.; Sun G. Third-Order Nonlinear Plasmonic Materials: Enhancement and Limitations. Phys. Rev. A 2013, 88 (5), 053838 10.1103/PhysRevA.88.053838.

[ref110] Javani M. H.; Stockman M. I. Real and Imaginary Properties of Epsilon-Near-Zero Materials. Phys. Rev. Lett. 2016, 117 (10), 107404 10.1103/PhysRevLett.117.107404. 27636495

[ref111] Secondo R.; Khurgin J.; Kinsey N. Absorptive Loss and Band Non-Parabolicity as a Physical Origin of Large Nonlinearity in Epsilon-near-Zero Materials. Optical Materials Express 2020, 10 (7), 1545 10.1364/OME.394111.

[ref112] Hau L. V.; Harris S. E.; Dutton Z.; Behroozi C. H. Light Speed Reduction to 17 Metres per Second in an Ultracold Atomic Gas. Nature 1999, 397 (6720), 594–598. 10.1038/17561.

[ref113] Boller K. J.; Imamoglu A.; Harris S. E. Observation of Electromagnetically Induced Transparency. Phys. Rev. Lett. 1991, 66 (20), 2593 10.1103/PhysRevLett.66.2593. 10043562

[ref114] Boyd R. W. Material Slow Light and Structural Slow Light: Similarities and Differences for Nonlinear Optics [Invited]. Journal of the Optical Society of America B 2011, 28 (12), A38 10.1364/JOSAB.28.000A38.

[ref115] Khurgin J. B. Slow Light in Various Media: A Tutorial. Advances in Optics and Photonics 2010, 2 (3), 287 10.1364/AOP.2.000287.

[ref116] Maccaferri N.; Zhao Y.; Isoniemi T.; Iarossi M.; Parracino A.; Strangi G.; De Angelis F. Hyperbolic Meta-Antennas Enable Full Control of Scattering and Absorption of Light. Nano Lett. 2019, 19 (3), 1851–1859. 10.1021/acs.nanolett.8b04841. 30776244

[ref117] Isoniemi T.; Maccaferri N.; Ramasse Q. M.; Strangi G.; De Angelis F. Electron Energy Loss Spectroscopy of Bright and Dark Modes in Hyperbolic Metamaterial Nanostructures. Adv. Optical Mater. 2020, 8 (13), 2000277 10.1002/adom.202000277.

[ref118] Kuttruff J.; Gabbani A.; Petrucci G.; Zhao Y.; Iarossi M.; Pedrueza-Villalmanzo E.; Dmitriev A.; Parracino A.; Strangi G.; De Angelis F.; Brida D.; Pineider F.; Maccaferri N. Magneto-Optical Activity in Nonmagnetic Hyperbolic Nanoparticles. Phys. Rev. Lett. 2021, 127 (21), 217402 10.1103/PhysRevLett.127.217402. 34860084

[ref119] Maccaferri N.; Zilli A.; Isoniemi T.; Ghirardini L.; Iarossi M.; Finazzi M.; Celebrano M.; De Angelis F. Enhanced Nonlinear Emission from Single Multilayered Metal-Dielectric Nanocavities Resonating in the Near-Infrared. ACS Photonics 2021, 8 (2), 512–520. 10.1021/acsphotonics.0c01500.

[ref120] Dhama R.; Habib M.; Rashed A. R.; Caglayan H. Unveiling Long-Lived Hot-Electron Dynamics via Hyperbolic Meta-Antennas. Nano Lett. 2023, 23 (8), 3122–3127. 10.1021/acs.nanolett.2c03922. 36867120 PMC10141405

[ref121] Kuttruff J.; Garoli D.; Allerbeck J.; Krahne R.; De Luca A.; Brida D.; Caligiuri V.; Maccaferri N. Ultrafast All-Optical Switching Enabled by Epsilon-near-Zero-Tailored Absorption in Metal-Insulator Nanocavities. Communications Physics 2020, 3 (1), 114 10.1038/s42005-020-0379-2.

[ref122] Pianelli A.; Dhama R.; Judek J.; Mazur R.; Caglayan H. Two-Color All-Optical Switching in Si-Compatible Epsilon-near-Zero Hyperbolic Metamaterials. arXiv:2305.06731 [physics.optics] 2023, na 10.48550/ARXIV.2305.06731.

[ref123] Caligiuri V.; Pianelli A.; Miscuglio M.; Patra A.; Maccaferri N.; Caputo R.; De Luca A. Near- and Mid-Infrared Graphene-Based Photonic Architectures for Ultrafast and Low-Power Electro-Optical Switching and Ultra-High Resolution Imaging. ACS Appl. Nano Mater. 2020, 3 (12), 12218–12230. 10.1021/acsanm.0c02690.

[ref124] Bohn J.; Luk T. S.; Tollerton C.; Hutchings S. W.; Brener I.; Horsley S.; Barnes W. L.; Hendry E. All-Optical Switching of an Epsilon-near-Zero Plasmon Resonance in Indium Tin Oxide. Nat. Commun. 2021, 12 (1), 1017 10.1038/s41467-021-21332-y. 33589641 PMC7884677

[ref125] Yang Y.; Kelley K.; Sachet E.; Campione S.; Luk T. S.; Maria J.-P.; Sinclair M. B.; Brener I. Femtosecond Optical Polarization Switching Using a Cadmium Oxide-Based Perfect Absorber. Nature Photon 2017, 11 (6), 390–395. 10.1038/nphoton.2017.64.

[ref126] Vassant S.; Hugonin J.-P.; Marquier F.; Greffet J.-J. Berreman Mode and Epsilon near Zero Mode. Opt. Express 2012, 20 (21), 23971 10.1364/OE.20.023971. 23188363

[ref127] Li A.; Reutzel M.; Wang Z.; Novko D.; Gumhalter B.; Petek H. Plasmonic Photoemission from Single-Crystalline Silver. ACS Photonics 2021, 8 (1), 247–258. 10.1021/acsphotonics.0c01412.

[ref128] Reutzel M.; Li A.; Gumhalter B.; Petek H. Nonlinear Plasmonic Photoelectron Response of Ag(111). Phys. Rev. Lett. 2019, 123 (1), 017404 10.1103/PhysRevLett.123.017404. 31386417

[ref129] Bruno V.; DeVault C.; Vezzoli S.; Kudyshev Z.; Huq T.; Mignuzzi S.; Jacassi A.; Saha S.; Shah Y. D.; Maier S. A.; Cumming D. R. S.; Boltasseva A.; Ferrera M.; Clerici M.; Faccio D.; Sapienza R.; Shalaev V. M. Negative Refraction in Time-Varying Strongly Coupled Plasmonic-Antenna-Epsilon-Near-Zero Systems. Phys. Rev. Lett. 2020, 124 (4), 043902 10.1103/PhysRevLett.124.043902. 32058792

[ref130] Bruno V.; Vezzoli S.; DeVault C.; Carnemolla E.; Ferrera M.; Boltasseva A.; Shalaev V. M.; Faccio D.; Clerici M. Broad Frequency Shift of Parametric Processes in Epsilon-Near-Zero Time-Varying Media. Applied Sciences 2020, 10 (4), 1318 10.3390/app10041318.

[ref131] Suresh S.; Reshef O.; Alam M. Z.; Upham J.; Karimi M.; Boyd R. W. Enhanced Nonlinear Optical Responses of Layered Epsilon-near-Zero Metamaterials at Visible Frequencies. ACS Photonics 2021, 8 (1), 125–129. 10.1021/acsphotonics.0c01178.

[ref132] Hohlfeld J.; Wellershoff S. S.; Güdde J.; Conrad U.; Jähnke V.; Matthias E. Electron and Lattice Dynamics Following Optical Excitation of Metals. Chem. Phys. 2000, 251 (1), 237–258. 10.1016/S0301-0104(99)00330-4.

[ref133] Jaffray W.; Belli F.; Carnemolla E. G.; Dobas C.; Mackenzie M.; Travers J.; Kar A. K.; Clerici M.; DeVault C.; Shalaev V. M.; Boltasseva A.; Ferrera M. Near-Zero-Index Ultra-Fast Pulse Characterization. Nat. Commun. 2022, 13 (1), 3536 10.1038/s41467-022-31151-4. 35725983 PMC9209551

[ref134] Shaltout A.; Clerici M.; Kinsey N.; Kaipurath R.; Kim J.; Carnemolla E. G.; Faccio D.; Boltasseva A.; Shalaev V. M.; Ferrera M. Doppler-Shift Emulation Using Highly Time-Refracting TCO Layer. In Conference on Lasers and Electro-Optics, San Jose, California, U.S.A., June 5–10, 2016, OSA: Washington, D.C., 2016; p FF2D.6. 10.1364/CLEO_QELS.2016.FF2D.6.

[ref135] Pang K.; Alam M. Z.; Zhou Y.; Liu C.; Reshef O.; Manukyan K.; Voegtle M.; Pennathur A.; Tseng C.; Su X.; Song H.; Zhao Z.; Zhang R.; Song H.; Hu N.; Almaiman A.; Dawlaty J. M.; Boyd R. W.; Tur M.; Willner A. E. Adiabatic Frequency Conversion Using a Time-Varying Epsilon-Near-Zero Metasurface. Nano Lett. 2021, 21 (14), 5907–5913. 10.1021/acs.nanolett.1c00550. 34251831

[ref136] Bruno V.; Devault C.; Vezzoli S.; Kudyshev Z.; Huq T.; Mignuzzi S.; Jacassi A.; Saha S.; Shah Y. D.; Maier S. A.; Cumming D. R. S.; Boltasseva A.; Ferrera M.; Clerici M.; Faccio D.; Sapienza R.; Shalaev V. M. Negative Refraction in Time-Varying Strongly Coupled Plasmonic-Antenna-Epsilon-Near-Zero Systems. Phys. Rev. Lett. 2020, 124 (4), 043902 10.1103/PhysRevLett.124.043902. 32058792

[ref137] Gosciniak J.; Hu Z.; Thomaschewski M.; Sorger V. J.; Khurgin J. B. Bistable All-Optical Devices Based on Nonlinear Epsilon-Near-Zero (ENZ) Materials. Laser & Photonics Reviews 2023, 17 (4), 2200723 10.1002/lpor.202200723.

[ref138] Wang R.; Hu F.; Meng Y.; Gong M.; Liu Q. High-Contrast Optical Bistability Using a Subwavelength Epsilon-near-Zero Material. Opt. Lett. 2023, 48 (6), 1371 10.1364/OL.481688. 36946930

[ref139] Clerici M.; Kinsey N.; DeVault C.; Kim J.; Carnemolla E. G.; Caspani L.; Shaltout A.; Faccio D.; Shalaev V.; Boltasseva A.; Ferrera M. Controlling Hybrid Nonlinearities in Transparent Conducting Oxides via Two-Colour Excitation. Nat. Commun. 2017, 8 (1), 15829 10.1038/ncomms15829. 28598441 PMC5472708

[ref140] Minerbi E.; Sideris S.; Khurgin J. B.; Ellenbogen T. The Role of Epsilon Near Zero and Hot Electrons in Enhanced Dynamic THz Emission from Nonlinear Metasurfaces. Nano Lett. 2022, 22 (15), 6194–6199. 10.1021/acs.nanolett.2c01400. 35899937 PMC9373027

[ref141] Altug H.; Oh S.-H.; Maier S. A.; Homola J. Advances and Applications of Nanophotonic Biosensors. Nat. Nanotechnol. 2022, 17 (1), 5–16. 10.1038/s41565-021-01045-5. 35046571

[ref142] Palermo G.; Sreekanth K. V.; Maccaferri N.; Lio G. E.; Nicoletta G.; De Angelis F.; Hinczewski M.; Strangi G. Hyperbolic Dispersion Metasurfaces for Molecular Biosensing. Nanophotonics 2020, 10 (1), 295–314. 10.1515/nanoph-2020-0466.

[ref143] Mejía-Salazar J. R.; Oliveira O. N. Plasmonic Biosensing. Chem. Rev. 2018, 118 (20), 10617–10625. 10.1021/acs.chemrev.8b00359. 30247025

[ref144] Kabashin A. V.; Evans P.; Pastkovsky S.; Hendren W.; Wurtz G. A.; Atkinson R.; Pollard R.; Podolskiy V. A.; Zayats A. V. Plasmonic Nanorod Metamaterials for Biosensing. Nat. Mater. 2009, 8, 867 10.1038/nmat2546. 19820701

[ref145] Yan R.; Wang T.; Yue X.; Wang H.; Zhang Y.-H.; Xu P.; Wang L.; Wang Y.; Zhang J. Highly Sensitive Plasmonic Nanorod Hyperbolic Metamaterial Biosensor. Photon. Res. 2022, 10 (1), 84 10.1364/PRJ.444490.

[ref146] Sreekanth K. V.; Alapan Y.; ElKabbash M.; Ilker E.; Hinczewski M.; Gurkan U. A.; De Luca A.; Strangi G. Extreme Sensitivity Biosensing Platform Based on Hyperbolic Metamaterials. Nat. Mater. 2016, 15 (6), 621–627. 10.1038/nmat4609. 27019384 PMC4959915

[ref147] Avrutsky I.; Salakhutdinov I.; Elser J.; Podolskiy V. Highly Confined Optical Modes in Nanoscale Metal-Dielectric Multilayers. Phys. Rev. B 2007, 75 (24), 241402 10.1103/PhysRevB.75.241402.

[ref148] Maccaferri N.; Isoniemi T.; Hinczewski M.; Iarossi M.; Strangi G.; De Angelis F. Designer Bloch Plasmon Polariton Dispersion in Grating-Coupled Hyperbolic Metamaterials. APL Photonics 2020, 5 (7), 076109 10.1063/5.0008687.

[ref149] Díaz-Valencia B. F.; Porras-Montenegro N.; Oliveira O. N.; Mejía-Salazar J. R. Nanostructured Hyperbolic Metamaterials for Magnetoplasmonic Sensors. ACS Appl. Nano Mater. 2022, 5 (2), 1740–1744. 10.1021/acsanm.1c04310.

[ref150] Indukuri S. R. K. C.; Frydendahl C.; Sharma N.; Mazurski N.; Paltiel Y.; Levy U. Enhanced Chiral Sensing at the Few-Molecule Level Using Negative Index Metamaterial Plasmonic Nanocuvettes. ACS Nano 2022, 16 (10), 17289–17297. 10.1021/acsnano.2c08090. 36194513

[ref151] Palermo G.; Lio G. E.; Esposito M.; Ricciardi L.; Manoccio M.; Tasco V.; Passaseo A.; De Luca A.; Strangi G. Biomolecular Sensing at the Interface between Chiral Metasurfaces and Hyperbolic Metamaterials. ACS Appl. Mater. Interfaces 2020, 12 (27), 30181–30188. 10.1021/acsami.0c07415. 32551524

[ref152] Wang X.; Choi J.; Liu J.; Malis O.; Li X.; Bermel P.; Zhang X.; Wang H. 3D Hybrid Trilayer Heterostructure: Tunable Au Nanorods and Optical Properties. ACS Appl. Mater. Interfaces 2020, 12 (40), 45015–45022. 10.1021/acsami.0c14937. 32960570

[ref153] Lee M.; Lee E.; So S.; Byun S.; Son J.; Ge B.; Lee H.; Park H. S.; Shim W.; Pee J. H.; Min B.; Cho S.-P.; Shi Z.; Noh T. W.; Rho J.; Kim J.-Y.; Chung I. Bulk Metamaterials Exhibiting Chemically Tunable Hyperbolic Responses. J. Am. Chem. Soc. 2021, 143 (49), 20725–20734. 10.1021/jacs.1c08446. 34783563

[ref154] Fusco Z.; Taheri M.; Bo R.; Tran-Phu T.; Chen H.; Guo X.; Zhu Y.; Tsuzuki T.; White T. P.; Tricoli A. Non-Periodic Epsilon-Near-Zero Metamaterials at Visible Wavelengths for Efficient Non-Resonant Optical Sensing. Nano Lett. 2020, 20 (5), 3970–3977. 10.1021/acs.nanolett.0c01095. 32343590

[ref155] Fan B.; Nasir M. E.; Nicholls L. H.; Zayats A. V.; Podolskiy V. A. Magneto-Optical Metamaterials: Nonreciprocal Transmission and Faraday Effect Enhancement. Adv. Optical Mater. 2019, 7 (14), 1801420 10.1002/adom.201801420.

[ref156] Kolmychek I. A.; Pomozov A. R.; Leontiev A. P.; Napolskii K. S.; Murzina T. V. Magneto-Optical Effects in Hyperbolic Metamaterials. Opt. Lett. 2018, 43 (16), 3917 10.1364/OL.43.003917. 30106916

[ref157] Malysheva I. V.; Kolmychek I. A.; Romashkina A. M.; Leontiev A. P.; Napolskii K. S.; Murzina T. V. Magneto-Optical Effects in Hyperbolic Metamaterials Based on Ordered Arrays of Bisegmented Gold/Nickel Nanorods. Nanotechnology 2021, 32 (30), 305710 10.1088/1361-6528/abf691. 33836510

[ref158] Wang X.; Jian J.; Wang H.; Liu J.; Pachaury Y.; Lu P.; Rutherford B. X.; Gao X.; Xu X.; El-Azab A.; Zhang X.; Wang H. Nitride-Oxide-Metal Heterostructure with Self-Assembled Core-Shell Nanopillar Arrays: Effect of Ordering on Magneto-Optical Properties. Small 2021, 17 (5), 2007222 10.1002/smll.202007222. 33448118

[ref159] Wang X.; Wang H.; Jian J.; Rutherford B. X.; Gao X.; Xu X.; Zhang X.; Wang H. Metal-Free Oxide-Nitride Heterostructure as a Tunable Hyperbolic Metamaterial Platform. Nano Lett. 2020, 20 (9), 6614–6622. 10.1021/acs.nanolett.0c02440. 32787175

[ref160] Bonanni V.; Bonetti S.; Pakizeh T.; Pirzadeh Z.; Chen J.; Nogués J.; Vavassori P.; Hillenbrand R.; Åkerman J.; Dmitriev A. Designer Magnetoplasmonics with Nickel Nanoferromagnets. Nano Lett. 2011, 11 (12), 5333–5338. 10.1021/nl2028443. 22029387 PMC3238448

[ref161] Maccaferri N.; E. Gregorczyk K.; de Oliveira T. V. A. G.; Kataja M.; van Dijken S.; Pirzadeh Z.; Dmitriev A.; Åkerman J.; Knez M.; Vavassori P. Ultrasensitive and Label-Free Molecular-Level Detection Enabled by Light Phase Control in Magnetoplasmonic Nanoantennas. Nat. Commun. 2015, 6 (1), 6150 10.1038/ncomms7150. 25639190 PMC4340560

[ref162] Manera M. G.; Colombelli A.; Taurino A.; Martin A. G.; Rella R. Magneto-Optical Properties of Noble-Metal Nanostructures: Functional Nanomaterials for Bio Sensing. Sci. Rep 2018, 8 (1), 12640 10.1038/s41598-018-30862-3. 30139943 PMC6107575

[ref163] Pourjamal S.; Kataja M.; Maccaferri N.; Vavassori P.; van Dijken S. Hybrid Ni/SiO_2_/Au Dimer Arrays for High-Resolution Refractive Index Sensing. Nanophotonics 2018, 7 (5), 905–912. 10.1515/nanoph-2018-0013.

[ref164] Pineider F.; Campo G.; Bonanni V.; de Julián Fernández C.; Mattei G.; Caneschi A.; Gatteschi D.; Sangregorio C. Circular Magnetoplasmonic Modes in Gold Nanoparticles. Nano Lett. 2013, 13 (10), 4785–4789. 10.1021/nl402394p. 24050533

[ref165] Rizal C.; Manera M. G.; Ignatyeva D. O.; Mejía-Salazar J. R.; Rella R.; Belotelov V. I.; Pineider F.; Maccaferri N. Magnetophotonics for Sensing and Magnetometry toward Industrial Applications. J. Appl. Phys. 2021, 130 (23), 230901 10.1063/5.0072884.

[ref166] Maccaferri N.; Gabbani A.; Pineider F.; Kaihara T.; Tapani T.; Vavassori P. Magnetoplasmonics in Confined Geometries: Current Challenges and Future Opportunities. Appl. Phys. Lett. 2023, 122 (12), 120502 10.1063/5.0136941.

[ref167] De Figueiredo F. A. P.; Moncada-Villa E.; Mejía-Salazar J. R. Optimization of Magnetoplasmonic ε-Near-Zero Nanostructures Using a Genetic Algorithm. Sensors 2022, 22 (15), 5789 10.3390/s22155789. 35957345 PMC9371128

[ref168] Galiffi E.; Tirole R.; Yin S.; Li H.; Vezzoli S.; Huidobro P. A.; Silveirinha M. G.; Sapienza R.; Alù A.; Pendry J. B. Photonics of Time-Varying Media. Adv. Photon. 2022, 4 (01), 014002 10.1117/1.AP.4.1.014002.

[ref169] Yu Z.; Fan S. Complete Optical Isolation Created by Indirect Interband Photonic Transitions. Nature Photon 2009, 3 (2), 91–94. 10.1038/nphoton.2008.273.

[ref170] Lira H.; Yu Z.; Fan S.; Lipson M. Electrically Driven Nonreciprocity Induced by Interband Photonic Transition on a Silicon Chip. Phys. Rev. Lett. 2012, 109 (3), 033901 10.1103/PhysRevLett.109.033901. 22861851

[ref171] Estep N. A.; Sounas D. L.; Soric J.; Alù A. Magnetic-Free Non-Reciprocity and Isolation Based on Parametrically Modulated Coupled-Resonator Loops. Nature Phys. 2014, 10 (12), 923–927. 10.1038/nphys3134.

[ref172] Shaltout A.; Kildishev A.; Shalaev V. Time-Varying Metasurfaces and Lorentz Non-Reciprocity. Opt. Mater. Express 2015, 5 (11), 2459 10.1364/OME.5.002459.

[ref173] Sounas D. L.; Alù A. Non-Reciprocal Photonics Based on Time Modulation. Nat. Photonics 2017, 11 (12), 774–783. 10.1038/s41566-017-0051-x.

[ref174] Huidobro P. A.; Galiffi E.; Guenneau S.; Craster R. V.; Pendry J. B. Fresnel Drag in Space-Time-Modulated Metamaterials. Proc. Natl. Acad. Sci. U.S.A. 2019, 116 (50), 24943–24948. 10.1073/pnas.1915027116. 31767741 PMC6911182

[ref175] Karl N.; Vabishchevich P. P.; Shcherbakov M. R.; Liu S.; Sinclair M. B.; Shvets G.; Brener I. Frequency Conversion in a Time-Variant Dielectric Metasurface. Nano Lett. 2020, 20 (10), 7052–7058. 10.1021/acs.nanolett.0c02113. 32940476

[ref176] Shcherbakov M. R.; Werner K.; Fan Z.; Talisa N.; Chowdhury E.; Shvets G. Photon Acceleration and Tunable Broadband Harmonics Generation in Nonlinear Time-Dependent Metasurfaces. Nat. Commun. 2019, 10 (1), 1345 10.1038/s41467-019-09313-8. 30902994 PMC6430811

[ref177] Zhou Y.; Alam M. Z.; Karimi M.; Upham J.; Reshef O.; Liu C.; Willner A. E.; Boyd R. W. Broadband Frequency Translation through Time Refraction in an Epsilon-near-Zero Material. Nat. Commun. 2020, 11 (1), 2180 10.1038/s41467-020-15682-2. 32358528 PMC7195366

[ref178] Tirole R.; Vezzoli S.; Galiffi E.; Robertson I.; Maurice D.; Tilmann B.; Maier S. A.; Pendry J. B.; Sapienza R. Double-Slit Time Diffraction at Optical Frequencies. Nat. Phys. 2023, 19 (7), 999–1002. 10.1038/s41567-023-01993-w.

[ref179] Fang K.; Yu Z.; Fan S. Realizing Effective Magnetic Field for Photons by Controlling the Phase of Dynamic Modulation. Nature Photon 2012, 6 (11), 782–787. 10.1038/nphoton.2012.236.

[ref180] Dutt A.; Lin Q.; Yuan L.; Minkov M.; Xiao M.; Fan S. A Single Photonic Cavity with Two Independent Physical Synthetic Dimensions. Science 2020, 367 (6473), 59–64. 10.1126/science.aaz3071. 31780626

[ref181] Yin S.; Alù A. Efficient Phase Conjugation in a Space-Time Leaky Waveguide. ACS Photonics 2022, 9 (3), 979–984. 10.1021/acsphotonics.1c01836.

[ref182] Liu T.; Ou J.-Y.; MacDonald K. F.; Zheludev N. I. Photonic Metamaterial Analogue of a Continuous Time Crystal. Nat. Phys. 2023, 19 (7), 986–991. 10.1038/s41567-023-02023-5.

[ref183] Moussa H.; Xu G.; Yin S.; Galiffi E.; Ra’di Y.; Alù A. Observation of Temporal Reflection and Broadband Frequency Translation at Photonic Time Interfaces. Nat. Phys. 2023, 19 (6), 863–868. 10.1038/s41567-023-01975-y.

[ref184] Galiffi E.; Xu G.; Yin S.; Moussa H.; Ra’di Y.; Alù A. Broadband Coherent Wave Control through Photonic Collisions at Time Interfaces. Nat. Phys. 2023, 10.1038/s41567-023-02165-6.

[ref185] Engheta N. Metamaterials with High Degrees of Freedom: Space, Time, and More. Nanophotonics 2020, 10 (1), 639–642. 10.1515/nanoph-2020-0414.

[ref186] Tirole R.; Galiffi E.; Dranczewski J.; Attavar T.; Tilmann B.; Wang Y.-T.; Huidobro P. A.; Alú A.; Pendry J. B.; Maier S. A.; Vezzoli S.; Sapienza R. Saturable Time-Varying Mirror Based on an Epsilon-Near-Zero Material. Phys. Rev. Applied 2022, 18 (5), 054067 10.1103/PhysRevApplied.18.054067.

[ref187] Guo P.; Schaller R. D.; Ketterson J. B.; Chang R. P. H. Ultrafast Switching of Tunable Infrared Plasmons in Indium Tin Oxide Nanorod Arrays with Large Absolute Amplitude. Nat. Photonics 2016, 10 (4), 267–273. 10.1038/nphoton.2016.14.

[ref188] Prain A.; Vezzoli S.; Westerberg N.; Roger T.; Faccio D. Spontaneous Photon Production in Time-Dependent Epsilon-Near-Zero Materials. Phys. Rev. Lett. 2017, 118 (13), 133904 10.1103/PhysRevLett.118.133904. 28409947

[ref189] Moshinsky M. Diffraction in Time. Phys. Rev. 1952, 88 (3), 625–631. 10.1103/PhysRev.88.625.

[ref190] Un I.-W.; Sarkar S.; Sivan Y. Electronic-Based Model of the Optical Nonlinearity of Low-Electron-Density Drude Materials. Phys. Rev. Applied 2023, 19 (4), 044043 10.1103/PhysRevApplied.19.044043.

[ref191] Sarkar S.; Un I. W.; Sivan Y. Electronic and Thermal Response of Low-Electron-Density Drude Materials to Ultrafast Optical Illumination. Phys. Rev. Applied 2023, 19 (1), 014005 10.1103/PhysRevApplied.19.014005.

[ref192] Bohn J.; Luk T. S.; Tollerton C.; Hutchings S. W.; Brener I.; Horsley S.; Barnes W. L.; Hendry E. All-Optical Switching of an Epsilon-near-Zero Plasmon Resonance in Indium Tin Oxide. Nat. Commun. 2021, 12 (1), 1017 10.1038/s41467-021-21332-y. 33589641 PMC7884677

[ref193] Asadchy V.; Lamprianidis A. G.; Ptitcyn G.; Albooyeh M.; Rituraj; Karamanos T.; Alaee R.; Tretyakov S. A.; Rockstuhl C.; Fan S. Parametric Mie Resonances and Directional Amplification in Time-Modulated Scatterers. Phys. Rev. Applied 2022, 18 (5), 054065 10.1103/PhysRevApplied.18.054065.

[ref194] Horsley S. A. R.; Galiffi E.; Wang Y.-T. Eigenpulses of Dispersive Time-Varying Media. Phys. Rev. Lett. 2023, 130 (20), 203803 10.1103/PhysRevLett.130.203803. 37267534

[ref195] Garg P.; Lamprianidis A. G.; Beutel D.; Karamanos T.; Verfürth B.; Rockstuhl C. Modeling Four-Dimensional Metamaterials: A T-Matrix Approach to Describe Time-Varying Metasurfaces. Opt. Express 2022, 30 (25), 45832 10.1364/OE.476035. 36522979

[ref196] Solís D. M.; Kastner R.; Engheta N. Time-Varying Materials in the Presence of Dispersion: Plane-Wave Propagation in a Lorentzian Medium with Temporal Discontinuity. Photon. Res. 2021, 9 (9), 1842 10.1364/PRJ.427368.

[ref197] Ortega-Gomez A.; Lobet M.; Vázquez-Lozano J. E.; Liberal I. Tutorial on the Conservation of Momentum in Photonic Time-Varying Media [Invited]. Opt. Mater. Express 2023, 13 (6), 1598 10.1364/OME.485540.

[ref198] Lyubarov M.; Lumer Y.; Dikopoltsev A.; Lustig E.; Sharabi Y.; Segev M. Amplified Emission and Lasing in Photonic Time Crystals. Science 2022, 377 (6604), 425–428. 10.1126/science.abo3324. 35679355

[ref199] Sharabi Y.; Dikopoltsev A.; Lustig E.; Lumer Y.; Segev M. Spatiotemporal Photonic Crystals. Optica 2022, 9 (6), 585 10.1364/OPTICA.455672.

[ref200] Galiffi E.; Huidobro P. A.; Pendry J. B. Broadband Nonreciprocal Amplification in Luminal Metamaterials. Phys. Rev. Lett. 2019, 123 (20), 206101 10.1103/PhysRevLett.123.206101. 31809075

[ref201] Pendry J. B.; Galiffi E.; Huidobro P. A. Gain Mechanism in Time-Dependent Media. Optica 2021, 8 (5), 636 10.1364/OPTICA.425582.

[ref202] Galiffi E.; Huidobro P. A.; Pendry J. B. An Archimedes’ Screw for Light. Nat. Commun. 2022, 13 (1), 2523 10.1038/s41467-022-30079-z. 35534459 PMC9085788

[ref203] Lustig E.; Sharabi Y.; Segev M. Topological Aspects of Photonic Time Crystals. Optica 2018, 5 (11), 1390 10.1364/OPTICA.5.001390.

[ref204] Galiffi E.; Wang Y.-T.; Lim Z.; Pendry J. B.; Alù A.; Huidobro P. A. Wood Anomalies and Surface-Wave Excitation with a Time Grating. Phys. Rev. Lett. 2020, 125 (12), 127403 10.1103/PhysRevLett.125.127403. 33016739

[ref205] Bugler-Lamb S.; Horsley S. A. R. Polariton Excitation Rates from Time Dependent Dielectrics. J. Phys. B: At. Mol. Opt. Phys. 2016, 49 (23), 235502 10.1088/0953-4075/49/23/235502.

[ref206] Sloan J.; Rivera N.; Joannopoulos J. D.; Soljačić M. Casimir Light in Dispersive Nanophotonics. Phys. Rev. Lett. 2021, 127 (5), 053603 10.1103/PhysRevLett.127.053603. 34397241

[ref207] Sloan J.; Rivera N.; Joannopoulos J. D.; Soljačić M. Controlling Two-Photon Emission from Superluminal and Accelerating Index Perturbations. Nat. Phys. 2022, 18 (1), 67–74. 10.1038/s41567-021-01428-4.

[ref208] Kort-Kamp W. J. M.; Azad A. K.; Dalvit D. A. R. Space-Time Quantum Metasurfaces. Phys. Rev. Lett. 2021, 127 (4), 043603 10.1103/PhysRevLett.127.043603. 34355970

[ref209] Dikopoltsev A.; Sharabi Y.; Lyubarov M.; Lumer Y.; Tsesses S.; Lustig E.; Kaminer I.; Segev M. Light Emission by Free Electrons in Photonic Time-Crystals. Proc. Natl. Acad. Sci. U.S.A. 2022, 119 (6), e2119705119 10.1073/pnas.2119705119. 35131857 PMC8833186

[ref210] Liu T.; Guo C.; Li W.; Fan S. Thermal Photonics with Broken Symmetries. eLight 2022, 2 (1), 25 10.1186/s43593-022-00025-z.

[ref211] Joulain K.; Mulet J.-P.; Marquier F.; Carminati R.; Greffet J.-J. Surface Electromagnetic Waves Thermally Excited: Radiative Heat Transfer, Coherence Properties and Casimir Forces Revisited in the near Field. Surf. Sci. Rep. 2005, 57 (3–4), 59–112. 10.1016/j.surfrep.2004.12.002.

[ref212] Greffet J.-J.; Bouchon P.; Brucoli G.; Marquier F. Light Emission by Nonequilibrium Bodies: Local Kirchhoff Law. Phys. Rev. X 2018, 8 (2), 021008 10.1103/PhysRevX.8.021008.

[ref213] Vázquez-Lozano J. E.; Liberal I. Incandescent Temporal Metamaterials. Nat. Commun. 2023, 14 (1), 4606 10.1038/s41467-023-40281-2. 37528085 PMC10394077

[ref214] Buddhiraju S.; Li W.; Fan S. Photonic Refrigeration from Time-Modulated Thermal Emission. Phys. Rev. Lett. 2020, 124 (7), 077402 10.1103/PhysRevLett.124.077402. 32142345

[ref215] Oh S.-H.; Altug H. Performance Metrics and Enabling Technologies for Nanoplasmonic Biosensors. Nat. Commun. 2018, 9 (1), 5263 10.1038/s41467-018-06419-3. 30531967 PMC6288137

[ref216] Maccaferri N.; Barbillon G.; Koya A. N.; Lu G.; Acuna G. P.; Garoli D. Recent Advances in Plasmonic Nanocavities for Single-Molecule Spectroscopy. Nanoscale Advances 2021, 3 (3), 633–642. 10.1039/D0NA00715C. 36133836 PMC9418431

[ref217] Li W.; Zhou J.; Maccaferri N.; Krahne R.; Wang K.; Garoli D. Enhanced Optical Spectroscopy for Multiplexed DNA and Protein-Sequencing with Plasmonic Nanopores: Challenges and Prospects. Anal. Chem. 2022, 94 (2), 503–514. 10.1021/acs.analchem.1c04459. 34974704 PMC8771637

[ref218] Kwaaitaal M.; Lourens D. G.; Davies C. S.; Kirilyuk A. Epsilon-near-Zero Regime as the Key to Ultrafast Control of Functional Properties of Solids. 19 May. arXiv:2305.11714 [cond-mat.mtrl-sci] 2023, na 10.48550/ARXIV.2305.11714.

